# Supplementary material for: Gender inequality in work location, childcare and work-life balance: Phase-specific differences throughout the COVID-19 pandemic
Source: PLoS One. 2024 Jun 25;19(6):e0302633. doi: 10.1371/journal.pone.0302633 (PMC11198899; doi:10.1371/journal.pone.0302633)

S1 Fig. Timeline of data collection in relation to relevant pandemic measures in the Netherlands.

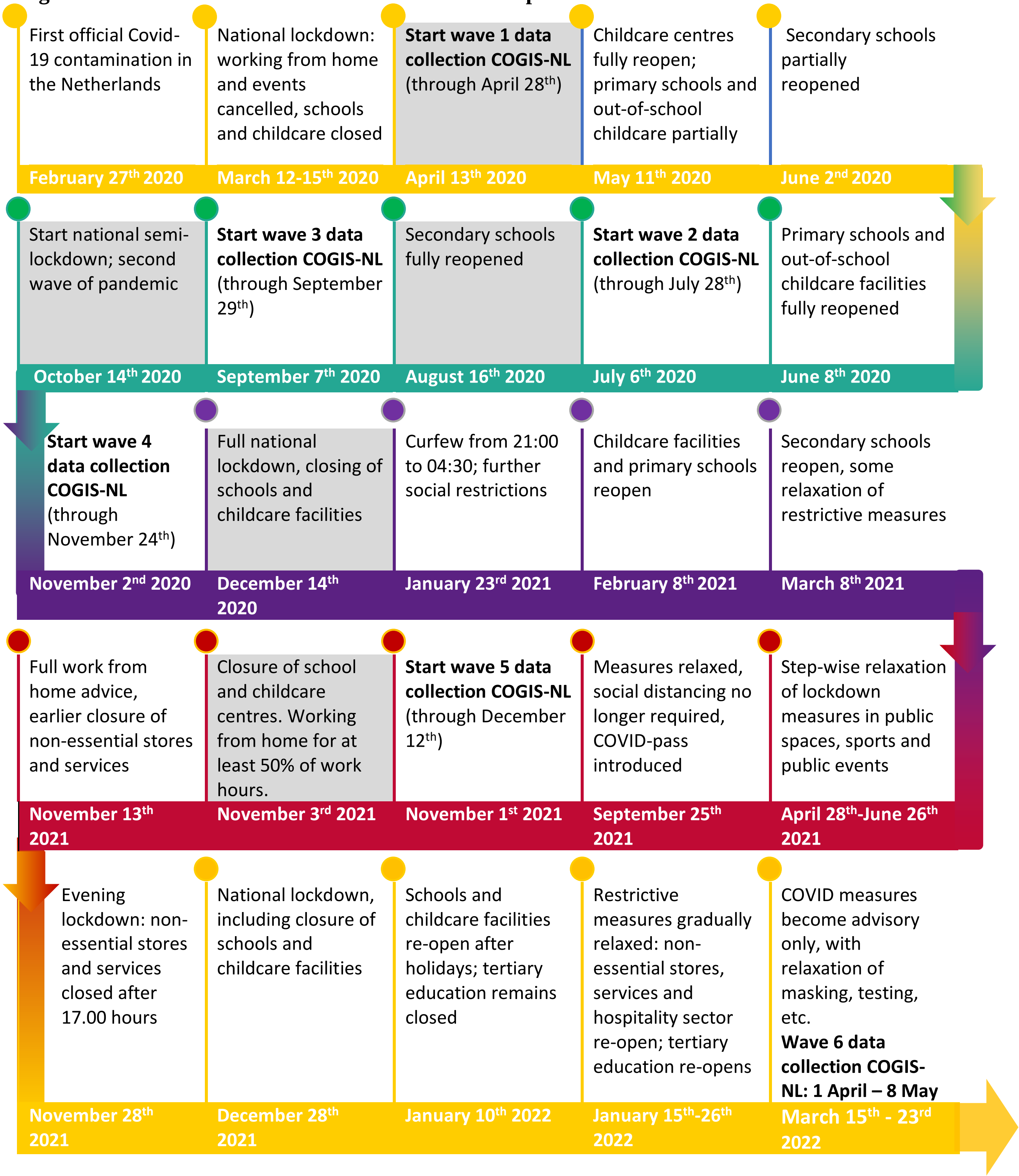

Supplement: S1 Fig — (PDF) [file pone.0302633.s001.pdf]
