# Supplementary material for: Gender inequality in work location, childcare and work-life balance: Phase-specific differences throughout the COVID-19 pandemic
Source: PLoS One. 2024 Jun 25;19(6):e0302633. doi: 10.1371/journal.pone.0302633 (PMC11198899; doi:10.1371/journal.pone.0302633)
Supplement: S1 Table — N/A: variable not measured in that wave; 0 = no missings. (DOCX) [file pone.0302633.s002.docx]

**S1 Table. Excluded cases by wave based on sample selection and missing values (N).**

|  | W1 | W2 | W3 | W4 | W5 | W6 |
| --- | --- | --- | --- | --- | --- | --- |
|  | Apr-20 | Jun-20 | Sept-20 | Nov-20 | Nov 21 | Apr-22 |
| **Sample selection variables** | | | | | | |
| No paid employment | 104 | 85 | 88 | 87 | 98 | 90 |
| No partner | 70 | 311 | 306 | 263 | 256 | 245 |
| **Total N after selection** | **680** | **828** | **851** | **740** | **746** | **704** |
|  |  |  |  |  |  |  |
| **Covariates** | | | | | | |
| Missings essential occupation | 0 | 1 | 0 | 2 | 27 | N/A |
| Missings partner in essential occupation | 0 | 2 | 7 | 7 | 0 | N/A |
| Missings age | 0 | 0 | 0 | 0 | 0 | 0 |
| Missings education level | 0 | 0 | 0 | 0 | 0 | 0 |
| Missings children | N/A | 0 | 0 | 0 | 0 | 0 |
| Missings work location autonomy | N/A | 1 | 2 | 4 | 0 | 0 |
| Missings work location partner | 35 | 37 | 32 | 20 | 20 | 13 |
| *Total cases deleted based on missing values* | *35* | *37* | *32* | *21* | *29* | *13* |
| **Total N after deletion:** | **645** | **791** | **819** | **719** | **717** | **691** |

N/A: variable not measured in that wave; 0=no missings.
