# Supplementary material for: Gender inequality in work location, childcare and work-life balance: Phase-specific differences throughout the COVID-19 pandemic
Source: PLoS One. 2024 Jun 25;19(6):e0302633. doi: 10.1371/journal.pone.0302633 (PMC11198899; doi:10.1371/journal.pone.0302633)
Supplement: S2 Table — (DOCX) [file pone.0302633.s003.docx]

**S2 Table. Descriptive statistics by wave.**

|  | W1  Apr-20 | | W2  Jun-20 | | W3  Sep-20 | | W4  Nov-20 | | W5  Nov-21 | | W6  Apr-22 | |
| --- | --- | --- | --- | --- | --- | --- | --- | --- | --- | --- | --- | --- |
|  | N | %/mean | N | %/mean | N | %/mean | N | %/mean | N | %/mean | N | %/mean |
| **Gender** |  |  |  |  |  |  |  |  |  |  |  |  |
| Female | 334 | 51.78 | 401 | 50.70 | 417 | 50.92 | 359 | 49.93 | 368 | 51.32 | 359 | 51.95 |
| Male | 311 | 48.22 | 390 | 49.30 | 402 | 49.08 | 360 | 50.07 | 349 | 48.68 | 332 | 48.05 |
| **Essential** **occupation** | |  |  |  |  |  |  |  |  |  |  |  |
| Yes | 295 | 45.74 | 341 | 43.11 | 354 | 43.22 | 335 | 46.59 | 325 | 45.33 | N/A | N/A |
| No | 350 | 54.26 | 450 | 56.89 | 465 | 56.78 | 384 | 53.41 | 392 | 54.67 | N/A | N/A |
| **Partner in essential occupation** | |  |  |  |  |  |  |  |  |  |  |  |
| Yes | 235 | 36.43 | 248 | 31.35 | 254 | 31.01 | 259 | 36.02 | 251 | 35.01 | N/A | N/A |
| No | 410 | 63.57 | 543 | 68.65 | 565 | 68.99 | 460 | 63.98 | 466 | 64.99 | N/A | N/A |
| Age | 645 | 42.65 | 791 | 43.01 | 819 | 43.39 | 719 | 43.42 | 717 | 44.92 | 691 | 45.20 |
| **Education level** |  |  |  |  |  |  |  |  |  |  |  |  |
| Primary | 64 | 9.92 | 89 | 11.25 | 103 | 12.58 | 98 | 13.63 | 91 | 12.69 | 81 | 11.72 |
| Secondary | 195 | 30.23 | 228 | 28.82 | 230 | 28.08 | 213 | 29.62 | 211 | 29.43 | 202 | 29.23 |
| Tertiary | 386 | 59.84 | 474 | 59.92 | 486 | 59.34 | 408 | 56.75 | 415 | 57.88 | 408 | 59.04 |
| **Co-resident minor child** |  |  |  |  |  |  |  |  |  |  |  |  |
| No | N/A | N/A | 260 | 32.87 | 266 | 32.48 | 228 | 31.71 | 214 | 29.85 | 198 | 28.65 |
| Yes | N/A | N/A | 531 | 67.13 | 553 | 67.52 | 491 | 68.29 | 503 | 70.15 | 493 | 71.35 |
| **Workplace autonomy** | |  |  |  |  |  |  |  |  |  |  |  |
| Agree | N/A | N/A | 410 | 51.83 | 405 | 49.45 | 417 | 58.00 | 346 | 48.26 | 293 | 42.40 |
| Neutral | N/A | N/A | 31 | 3.92 | 36 | 4.40 | 28 | 3.89 | 24 | 3.35 | 27 | 3.91 |
| Disagree | N/A | N/A | 260 | 32.87 | 292 | 35.65 | 206 | 28.65 | 258 | 35.98 | 265 | 38.35 |
| Not applicable | N/A | N/A | 90 | 11.38 | 86 | 10.50 | 68 | 9.46 | 89 | 12.41 | 106 | 15.34 |
| **Work location partner** | |  |  |  |  |  |  |  |  |  |  |  |
| Fully from home | 248 | 38.45 | 235 | 29.71 | 183 | 22.34 | 200 | 27.82 | 124 | 17.29 | 138 | 19.97 |
| Partially from home | 82 | 12.71 | 98 | 12.39 | 121 | 14.77 | 77 | 10.71 | 132 | 18.41 | 64 | 9.26 |
| Workplace – can work from home | 35 | 5.43 | 64 | 8.09 | 95 | 11.60 | 59 | 8.21 | 59 | 8.23 | 318 | 46.02 |
| Workplace - nature of work | 196 | 30.39 | 275 | 34.77 | 308 | 37.61 | 281 | 39.08 | 307 | 42.82 | 89 | 12.88 |
| Not employed | 84 | 13.02 | 119 | 15.04 | 112 | 13.68 | 102 | 14.19 | 95 | 13.25 | 89 | 12.88 |
