# Supplementary material for: Gender inequality in work location, childcare and work-life balance: Phase-specific differences throughout the COVID-19 pandemic
Source: PLoS One. 2024 Jun 25;19(6):e0302633. doi: 10.1371/journal.pone.0302633 (PMC11198899; doi:10.1371/journal.pone.0302633)
Supplement: S3 Table — (DOCX) [file pone.0302633.s004.docx]

**S3 Table. Excluded cases by wave (N) – dependent variables.**

|  | W1 | W2 | W3 | W4 | W5 | W6 |
| --- | --- | --- | --- | --- | --- | --- |
|  | Apr-20 | Jun-20 | Sept-20 | Nov-20 | Nov 21 | Apr-22 |
| *Sample 1* |  |  |  |  |  |  |
| Missings work location | 28 | 27 | 21 | 17 | 8 | 10 |
| **Final N work location after deletion** | **617** | **764** | **798** | **702** | **709** | **681** |
| *Sample 2* |  |  |  |  |  |  |
| No minor co-resident children at home | N/A | 260 | 266 | 228 | 214 | 198 |
| Missings on age youngest child | 41 | 5 | 4 | 6 | 5 | 17 |
| Missings division of childcare | 3 | 4 | 6 | 8 | 20 | 32 |
| **Final N childcare after deletion** | **603** | **522** | **543** | **480** | **479** | **456** |
| *Sample 3* |  |  |  |  |  |  |
| Missings work-life balance | 4 | 91 | 82 | 86 | 83 | 63 |
| **Final N work-life balance after deletion** | **641** | **700** | **737** | **633** | **634** | **628** |
