# Supplementary material for: Gender inequality in work location, childcare and work-life balance: Phase-specific differences throughout the COVID-19 pandemic
Source: PLoS One. 2024 Jun 25;19(6):e0302633. doi: 10.1371/journal.pone.0302633 (PMC11198899; doi:10.1371/journal.pone.0302633)
Supplement: S4 Table — (DOCX) [file pone.0302633.s005.docx]

**S4 Table. Descriptive statistics of dependent variables (work location, division of childcare and work-life balance), by wave.**

|  | W1  Apr-20 | | W2  Jun-20 | | W3  Sept-20 | | W4  Nov-20 | | W5  Nov 21 | | W6  Apr-22 | |
| --- | --- | --- | --- | --- | --- | --- | --- | --- | --- | --- | --- | --- |
| **Work location** |  | | | | | | | | | | | |
| Fully from home | 281 | 45.54 | 250 | 32.72 | 191 | 23.93 | 217 | 30.91 | 136 | 19.18 | 89 | 13.07 |
| Partially from home | 73 | 11.83 | 127 | 16.62 | 126 | 15.79 | 87 | 12.39 | 138 | 19.46 | 159 | 23.35 |
| Workplace – can work from home | 43 | 6.97 | 76 | 9.95 | 130 | 16.29 | 84 | 11.97 | 84 | 11.85 | 115 | 16.89 |
| Workplace - nature of the work | 220 | 35.66 | 311 | 40.71 | 351 | 43.98 | 314 | 44.73 | 351 | 49.51 | 318 | 46.70 |
| Total | 617 | 100 | 764 | 100 | 798 | 100 | 702 | 100 | 709 | 100 | 681 | 100 |
| **Childcare compared to partner** |  | | | | | | | | | | | |
| More childcare | 101 | 16.75 | 119 | 22.80 | 96 | 17.68 | 90 | 18.75 | 100 | 20.88 | 87 | 19.08 |
| Same childcare | 394 | 65.34 | 247 | 47.32 | 282 | 51.93 | 256 | 53.33 | 221 | 46.14 | 228 | 50.00 |
| Less childcare | 108 | 17.91 | 156 | 29.89 | 165 | 30.39 | 134 | 27.92 | 158 | 32.99 | 141 | 30.92 |
| Total | 603 | 100 | 522 | 100 | 543 | 100 | 480 | 100 | 479 | 100 | 456 | 100 |
| **Work-life balance** |  | | | | | | | | | | | |
| Easy | 239 | 37.29 | 328 | 46.86 | 390 | 52.92 | 356 | 56.24 | 366 | 57.73 | 367 | 58.44 |
| Neutral | 208 | 32.45 | 230 | 32.86 | 259 | 35.14 | 221 | 34.91 | 183 | 28.86 | 183 | 29.14 |
| Difficult | 194 | 30.27 | 142 | 20.29 | 88 | 11.94 | 56 | 8.85 | 85 | 13.41 | 78 | 12.42 |
| Total | 641 | 100 | 700 | 100 | 737 | 100 | 633 | 100 | 634 | 100 | 628 | 100 |
