# Supplementary material for: Gender inequality in work location, childcare and work-life balance: Phase-specific differences throughout the COVID-19 pandemic
Source: PLoS One. 2024 Jun 25;19(6):e0302633. doi: 10.1371/journal.pone.0302633 (PMC11198899; doi:10.1371/journal.pone.0302633)
Supplement: S5 Table — Note: Work location and work-life balance based on first wave that included both people with and without children (wave 2), childcare based on wave 1. For pre-pandemic work-life balance there were 8 missing values. (DOCX) [file pone.0302633.s006.docx]

**S5 Table. Descriptive statistics of dependent variables (work location, division of childcare and work-life balance) prior to the pandemic, by gender.**

|  | Women | | Men | |
| --- | --- | --- | --- | --- |
|  | N | % | N | % |
| **Work location** |  |  |  |  |
| Always worked from home and that hasn’t changed | 35 | 9.07 | 31 | 8.20 |
| Working from home due to the pandemic | 88 | 22.80 | 96 | 25.40 |
| Working partially from home due to the pandemic | 54 | 13.99 | 73 | 19.31 |
| Working at Workplace – can work from home | 35 | 9.07 | 41 | 10.85 |
| Working at workplace due to the nature of the work | 174 | 45.08 | 137 | 36.24 |
| Total | 386 | 100.00 | 378 | 100.00 |
| **Childcare relative to partner prior to the pandemic** |  |  |  |  |
| Does (much) more childcare | 186 | 59.62 | 12 | 4.12 |
| Equal division of childcare | 114 | 36.54 | 103 | 35.40 |
| Does (much) less childcare | 12 | 3.85 | 176 | 60.48 |
| Total | 312 | 100.00 | 291 | 100.00 |
| **Work-life balance prior to the pandemic** |  |  |  |  |
| Easy | 172 | 48.31 | 196 | 58.33 |
| Neutral | 136 | 38.20 | 104 | 30.95 |
| Difficult | 48 | 13.48 | 36 | 10.71 |
| Total | 356 | 100.00 | 336 | 100.00 |

Note: Work location and work-life balance based on first wave that included both people with and without children (wave 2), childcare based on wave 1. For pre-pandemic work-life balance there were 8 missing values.
