# Supplementary material for: Gender inequality in work location, childcare and work-life balance: Phase-specific differences throughout the COVID-19 pandemic
Source: PLoS One. 2024 Jun 25;19(6):e0302633. doi: 10.1371/journal.pone.0302633 (PMC11198899; doi:10.1371/journal.pone.0302633)
Supplement: S6 Table — Note: Standard errors in parentheses. *** p<0.01, ** p<0.05, * p<0.1. Controlled for all co-variates. Reference categories are women, non-essential occupations, partner in non-essential occupation, vocational education, no minor co-resident children, neutral on statement ‘I can decide where I work’, partner working on location due to the nature of the work. (DOCX) [file pone.0302633.s007.docx]

**S6 Table. Marginal effect of gender on work location, with and without (w/o) minor, co-resident children.**

|  | Jun-20 | Sept-20 | Nov-20 | Nov 21 | Apr-22 |
| --- | --- | --- | --- | --- | --- |
|  | dy/dx | dy/dx | dy/dx | dy/dx | dy/dx |
| **Working from home** |  |  |  |  |  |
| Man w/o minor co-resident children (compared to woman w/o minor co-resident children) | -0.0281 | -0.0339 | -0.0938 | -0.0584 | -0.0347 |
|  | (0.0533) | (0.0504) | (0.0576) | (0.0521) | (0.0476) |
| Father (compared to Mother) | -0.0526 | -0.0161 | -0.0377 | -0.0481 | -0.0111 |
|  | (0.0384) | (0.0364) | (0.0395) | (0.0346) | (0.0294) |
| **Working partially from home** |  |  |  |  |  |
| Man w/o minor co-resident children (compared to woman w/o minor co-resident children) | 0.0904** | 0.0427 | -0.00303 | 0.0276 | 0.0248 |
|  | (0.0428) | (0.0405) | (0.0424) | (0.0473) | (0.0516) |
| Father (compared to Mother) | 0.0450 | -0.0165 | 0.00606 | -0.00749 | -0.0116 |
|  | (0.0335) | (0.0312) | (0.0289) | (0.0334) | (0.0348) |
| **Working at Workplace – can work from home** |  |  |  |  |  |
| Man w/o minor co-resident children (compared to woman w/o minor co-resident children) | 0.00143 | 0.0516 | 0.0442 | -0.0343 | -0.0310 |
|  | (0.0336) | (0.0446) | (0.0398) | (0.0431) | (0.0522) |
| Father (compared to Mother) | 0.0200 | 0.0583* | 0.0229 | 0.0177 | 0.0323 |
|  | (0.0286) | (0.0329) | (0.0320) | (0.0304) | (0.0343) |
| **Working at workplace due to the nature of the work** |  |  |  |  |  |
| Man w/o minor co-resident children (compared to woman w/o minor co-resident children) | -0.0637 | -0.0605 | 0.0526 | 0.0651 | 0.0409 |
|  | (0.0488) | (0.0486) | (0.0541) | (0.0487) | (0.0515) |
| Father (compared to Mother) | -0.0124 | -0.0257 | 0.00874 | 0.0378 | -0.00962 |
|  | (0.0360) | (0.0341) | (0.0395) | (0.0324) | (0.0337) |
| Observations | 764 | 798 | 702 | 709 | 681 |

Note: Standard errors in parentheses. *** p<0.01, ** p<0.05, * p<0.1. Controlled for all co-variates. Reference categories are women, non-essential occupations, partner in non-essential occupation, vocational education, no minor co-resident children, neutral on statement ‘I can decide where I work’, partner working on location due to the nature of the work.
