# Supplementary material for: Gender inequality in work location, childcare and work-life balance: Phase-specific differences throughout the COVID-19 pandemic
Source: PLoS One. 2024 Jun 25;19(6):e0302633. doi: 10.1371/journal.pone.0302633 (PMC11198899; doi:10.1371/journal.pone.0302633)
Supplement: S7 Table — Note: Standard errors in parentheses. *** p<0.01, ** p<0.05, * p<0.1. Controlled for all co-variates. Reference categories are women, non-essential occupations, partner in non-essential occupation, vocational education, no minor co-resident children, neutral on statement ‘I can decide where I work’, partner working on location due to the nature of the work. (DOCX) [file pone.0302633.s008.docx]

**S7 Table.** **Marginal effect of gender on work location in essential and non-essential occupations.**

|  | Apr-20 | Jun-20 | Sept-20 | Nov-20 | Nov 21 |
| --- | --- | --- | --- | --- | --- |
|  | dy/dx | dy/dx | dy/dx | dy/dx | dy/dx |
| **Fully from home** |  |  |  |  |  |
| Man non-essential (compared to woman non-essential) | -0.113** | -0.101** | -0.0464 | -0.129** | -0.120*** |
|  | (0.0508) | (0.0469) | (0.0443) | (0.0519) | (0.0431) |
| Man essential (compared to woman essential) | -0.0165 | 0.0238 | 0.00216 | 0.0160 | 0.0425 |
|  | (0.0543) | (0.0415) | (0.0387) | (0.0411) | (0.0399) |
| **Partially from home** |  |  |  |  |  |
| Man non-essential (compared to woman non-essential) | 0.0460 | 0.0307 | -0.00145 | -0.0257 | 0.00894 |
|  | (0.0329) | (0.0331) | (0.0327) | (0.0307) | (0.0349) |
| Man essential (compared to woman essential) | 0.0661 | 0.107** | 0.0143 | 0.0423 | -0.0104 |
|  | (0.0460) | (0.0459) | (0.0401) | (0.0416) | (0.0454) |
| **Working at Workplace – can work from home** |  |  |  |  |  |
| Man non-essential (compared to woman non-essential) | 0.0495** | 0.0216 | 0.0363 | 0.0439 | 0.0134 |
|  | (0.0223) | (0.0262) | (0.0344) | (0.0323) | (0.0337) |
| Man essential (compared to woman essential) | 0.0278 | -0.00297 | 0.0821* | 0.00903 | -0.0245 |
|  | (0.0395) | (0.0393) | (0.0452) | (0.0402) | (0.0386) |
| **Working at workplace due to the nature of the work** |  |  |  |  |  |
| Man non-essential (compared to woman non-essential) | 0.0177 | 0.0490 | 0.0115 | 0.111** | 0.0979** |
|  | (0.0449) | (0.0394) | (0.0392) | (0.0448) | (0.0412) |
| Man essential (compared to woman essential) | -0.0774 | -0.128*** | -0.0986** | -0.0674 | -0.00756 |
|  | (0.0546) | (0.0455) | (0.0433) | (0.0481) | (0.0397) |
| Observations | 617 | 764 | 798 | 702 | 709 |

Note: Standard errors in parentheses. *** p<0.01, ** p<0.05, * p<0.1. Controlled for all co-variates. Reference categories are women, non-essential occupations, partner in non-essential occupation, vocational education, no minor co-resident children, neutral on statement ‘I can decide where I work’, partner working on location due to the nature of the work.
