# Supplementary material for: Gender inequality in work location, childcare and work-life balance: Phase-specific differences throughout the COVID-19 pandemic
Source: PLoS One. 2024 Jun 25;19(6):e0302633. doi: 10.1371/journal.pone.0302633 (PMC11198899; doi:10.1371/journal.pone.0302633)
Supplement: S8 Table — Note: Standard errors in parentheses. *** p<0.01, ** p<0.05, * p<0.1. Controlled for all co-variates. Reference categories are women, non-essential occupations, partner in non-essential occupation, vocational education, no minor co-resident children, neutral on statement ‘I can decide where I work’, partner working on location due to the nature of the work. (DOCX) [file pone.0302633.s009.docx]

**S8 Table. Marginal effect of gender on work location across educational groups.**

|  | Apr-20 | Jun-20 | Sept-20 | Nov-20 | Nov 21 | Apr-22 |
| --- | --- | --- | --- | --- | --- | --- |
|  | dy/dx | dy/dx | dy/dx | dy/dx | dy/dx | dy/dx |
| **Fully from home** |  |  |  |  |  |  |
| Prim. / sec. educated man (vs prim. / sec. educated woman) | -0.170 | -0.264*** | -0.0365 | -0.166** | -0.0895 | -0.108 |
|  | (0.107) | (0.0858) | (0.0811) | (0.0742) | (0.0739) | (0.0803) |
| Vocational educated man (vs vocational educated woman) | -0.0485 | -0.0952* | -0.0497 | -0.0531 | -0.0779 | -0.0373 |
|  | (0.0666) | (0.0566) | (0.0544) | (0.0589) | (0.0534) | (0.0534) |
| Tertiary educated man (vs tertiary educated woman) | -0.0684 | 0.00561 | -0.0131 | -0.0406 | -0.0312 | -0.00112 |
|  | (0.0512) | (0.0429) | (0.0404) | (0.0469) | (0.0400) | (0.0325) |
| **Partially from home** |  |  |  |  |  |  |
| Prim. / sec. educated man (vs prim. / sec. educated woman) | 0.0363 | 0.171** | -0.00218 | 0.113* | 0.0632 | 0.178** |
|  | (0.0808) | (0.0739) | (0.0779) | (0.0674) | (0.0642) | (0.0813) |
| Vocational educated man (vs vocational educated woman) | 0.0423 | 0.117*** | 0.0792* | 0.0849* | 0.0720 | -0.0442 |
|  | (0.0551) | (0.0453) | (0.0436) | (0.0450) | (0.0551) | (0.0506) |
| Tertiary educated man (vs tertiary educated woman) | 0.0669* | 0.0366 | -0.0141 | -0.0370 | -0.0180 | -0.00302 |
|  | (0.0360) | (0.0357) | (0.0319) | (0.0299) | (0.0355) | (0.0387) |
| **Working at Workplace – can work from home** |  |  |  |  |  |  |
| Prim. / sec. educated man (vs prim. / sec. educated woman) | 0.0101 | -0.0881 | 0.0302 | -0.0169 | -0.180** | -0.146 |
|  | (0.0467) | (0.0811) | (0.0681) | (0.0669) | (0.0865) | (0.0936) |
| Vocational educated man (vs vocational educated woman) | 0.0393 | 0.0192 | 0.00911 | -0.0799 | -0.107** | 0.0713 |
|  | (0.0338) | (0.0468) | (0.0560) | (0.0497) | (0.0508) | (0.0592) |
| Tertiary educated man (vs tertiary educated woman) | 0.0498 | 0.0236 | 0.0780** | 0.0812** | 0.0694** | 0.0178 |
|  | (0.0334) | (0.0271) | (0.0346) | (0.0339) | (0.0314) | (0.0375) |
| **Working at workplace due to the nature of the work** |  |  |  |  |  |  |
| Prim. / sec. educated man (vs prim. / sec. educated woman) | 0.124 | 0.182** | 0.00850 | 0.0700 | 0.207*** | 0.0769 |
|  | (0.125) | (0.0907) | (0.0763) | (0.0875) | (0.0787) | (0.0855) |
| Vocational educated man (vs vocational educated woman) | -0.0331 | -0.0409 | -0.0387 | 0.0482 | 0.113** | 0.0102 |
|  | (0.0744) | (0.0566) | (0.0542) | (0.0603) | (0.0467) | (0.0532) |
| Tertiary educated man (vs tertiary educated woman) | -0.0483 | -0.0658* | -0.0508 | -0.00358 | -0.0202 | -0.0136 |
|  | (0.0438) | (0.0399) | (0.0385) | (0.0443) | (0.0396) | (0.0408) |
| Observations | 617 | 764 | 798 | 702 | 709 | 681 |

Note: Standard errors in parentheses. *** p<0.01, ** p<0.05, * p<0.1. Controlled for all co-variates. Reference categories are women, non-essential occupations, partner in non-essential occupation, vocational education, no minor co-resident children, neutral on statement ‘I can decide where I work’, partner working on location due to the nature of the work.
