# Supplementary material for: Gender inequality in work location, childcare and work-life balance: Phase-specific differences throughout the COVID-19 pandemic
Source: PLoS One. 2024 Jun 25;19(6):e0302633. doi: 10.1371/journal.pone.0302633 (PMC11198899; doi:10.1371/journal.pone.0302633)
Supplement: S9 Table — Note: Standard errors in parentheses. *** p<0.01, ** p<0.05, * p<0.1. Controlled for all co-variates. Reference categories are women, non-essential occupations, partner in non-essential occupation, vocational education, no minor co-resident children, neutral on statement ‘I can decide where I work’, partner working on location due to the nature of the work. Essential occupation was not measured in wave 6 (April 2022) and is therefore excluded from these analyses. (DOCX) [file pone.0302633.s010.docx]

**S9 Table. Marginal effect of gender on division of childcare tasks in essential and non-essential occupations.**

|  | Apr-20 | Jun-20 | Sept-20 | Nov-20 | Nov 21 |
| --- | --- | --- | --- | --- | --- |
|  | dy/dx | dy/dx | dy/dx | dy/dx | dy/dx |
| **More childcare** |  |  |  |  |  |
| Father non-essential (vs mother non-essential) | 0.0934* | 0.0711 | 0.1034** | -0.0380 | 0.0001 |
|  | (0.0482) | (0.0528) | (0.0453) | (0.0525) | (0.0520) |
| Father essential (vs mother essential) | 0.0801* | 0.1053* | 0.0486 | 0.0155 | -0.0282 |
|  | (0.0411) | (0.0602) | (0.0534) | (0.0581) | (0.0612) |
| **Same amount of childcare** |  |  |  |  |  |
| Father non-essential (vs mother non-essential) | -0.0225 | -0.0242 | 0.0438 | 0.2066*** | 0.1076* |
|  | (0.0552) | (0.0620) | (0.0611) | (0.0640) | (0.0648) |
| Father essential (vs mother essential) | -0.0433 | 0.0508 | 0.1692** | 0.0886 | 0.0910 |
|  | (0.0623) | (0.0718) | (0.0685) | (0.0699) | (0.0732) |
| **Less childcare** |  |  |  |  |  |
| Father non-essential (vs mother non-essential) | -0.0709* | -0.0469 | -0.1472** | -0.1687*** | -0.1077* |
|  | (0.0374) | (0.0571) | (0.0571) | (0.0604) | (0.0612) |
| Father essential (vs mother essential) | -0.0368 | -0.1561** | -0.2178*** | -0.1041* | -0.0628 |
|  | (0.0562) | (0.0644) | (0.0591) | (0.0629) | (0.0696) |
| Observations | 603 | 522 | 543 | 480 | 479 |

Note: Standard errors in parentheses. *** p<0.01, ** p<0.05, * p<0.1. Controlled for all co-variates. Reference categories are women, non-essential occupations, partner in non-essential occupation, vocational education, no minor co-resident children, neutral on statement ‘I can decide where I work’, partner working on location due to the nature of the work. Essential occupation was not measured in wave 6 (April 2022) and is therefore excluded from these analyses.
