# Supplementary material for: Gender inequality in work location, childcare and work-life balance: Phase-specific differences throughout the COVID-19 pandemic
Source: PLoS One. 2024 Jun 25;19(6):e0302633. doi: 10.1371/journal.pone.0302633 (PMC11198899; doi:10.1371/journal.pone.0302633)
Supplement: S10 Table — Note: Standard errors in parentheses. *** p<0.01, ** p<0.05, * p<0.1. Controlled for all co-variates. Reference categories are mothers, non-essential occupations, partner in non-essential occupation, vocational education, no minor co-resident children, neutral on statement ‘I can decide where I work’, partner working on location due to the nature of the work. (DOCX) [file pone.0302633.s011.docx]

**S10 Table. Marginal effect of gender on division of childcare tasks across educational groups.**

|  | Apr-20 | Jun-20 | Sept-20 | Nov-20 | Nov 21 | Apr-22 |
| --- | --- | --- | --- | --- | --- | --- |
|  | dy/dx | dy/dx | dy/dx | dy/dx | dy/dx | dy/dx |
| **More childcare** |  |  |  |  |  |  |
| Prim. / sec. educated father (vs prim. / sec. educated mother) | 0.107* | 0.0930 | 0.0927 | -0.0648 | -0.117 | 0.0579 |
|  | (0.0581) | (0.121) | (0.0992) | (0.0997) | (0.106) | (0.108) |
| Vocational educated father (vs vocational educated mother) | 0.127** | 0.0271 | 0.0229 | -0.0677 | -0.0441 | 0.0945 |
|  | (0.0509) | (0.0661) | (0.0639) | (0.0746) | (0.0689) | (0.0682) |
| Tertiary educated father (vs tertiary educated mother) | 0.0639 | 0.119** | 0.111** | 0.0267 | 0.0253 | 0.111** |
|  | (0.0443) | (0.0509) | (0.0440) | (0.0473) | (0.0527) | (0.0515) |
| **Same amount of childcare** |  |  |  |  |  |  |
| Prim. / sec. educated father (vs prim. / sec. educated mother) | -0.0430 | 0.0813 | 0.249** | 0.221* | 0.131 | 0.0551 |
|  | (0.130) | (0.132) | (0.125) | (0.126) | (0.140) | (0.139) |
| Vocational educated father (vs vocational educated mother) | -0.0575 | 0.0794 | 0.149* | 0.293*** | 0.180** | 0.0791 |
|  | (0.0705) | (0.0799) | (0.0795) | (0.0785) | (0.0828) | (0.0855) |
| Tertiary educated father (vs tertiary educated mother) | -0.0147 | -0.0432 | 0.0449 | 0.0584 | 0.0523 | -0.0246 |
|  | (0.0536) | (0.0613) | (0.0593) | (0.0623) | (0.0647) | (0.0653) |
| **Less childcare** |  |  |  |  |  |  |
| Prim. / sec. educated father (vs prim. / sec. educated mother) | -0.0638 | -0.174 | -0.342*** | -0.156 | -0.0144 | -0.113 |
|  | (0.126) | (0.140) | (0.126) | (0.132) | (0.135) | (0.137) |
| Vocational educated father (vs vocational educated mother) | -0.0691 | -0.106 | -0.172** | -0.225*** | -0.136* | -0.174** |
|  | (0.0582) | (0.0762) | (0.0746) | (0.0798) | (0.0800) | (0.0799) |
| Tertiary educated father (vs tertiary educated mother) | -0.0492 | -0.0757 | -0.156*** | -0.0851 | -0.0776 | -0.0866 |
|  | (0.0397) | (0.0536) | (0.0518) | (0.0524) | (0.0602) | (0.0577) |
| Observations | 603 | 522 | 543 | 480 | 479 | 456 |

Note: Standard errors in parentheses. *** p<0.01, ** p<0.05, * p<0.1. Controlled for all co-variates. Reference categories are mothers, non-essential occupations, partner in non-essential occupation, vocational education, no minor co-resident children, neutral on statement ‘I can decide where I work’, partner working on location due to the nature of the work.
