# Supplementary material for: Gender inequality in work location, childcare and work-life balance: Phase-specific differences throughout the COVID-19 pandemic
Source: PLoS One. 2024 Jun 25;19(6):e0302633. doi: 10.1371/journal.pone.0302633 (PMC11198899; doi:10.1371/journal.pone.0302633)
Supplement: S11 Table — Note: Standard errors in parentheses. *** p<0.01, ** p<0.05, * p<0.1. Controlled for all co-variates. Reference categories are women, non-essential occupations, partner in non-essential occupation, vocational education, no minor co-resident children, neutral on statement ‘I can decide where I work’, partner working on location due to the nature of the work. (DOCX) [file pone.0302633.s012.docx]

**S11 Table. Marginal effect of gender on work-life balance, with and without (w/o) minor co-resident children.**

|  | Jun-20 | Sept-20 | Nov-20 | Nov 21 | Apr-22 |
| --- | --- | --- | --- | --- | --- |
|  | dy/dx | dy/dx | dy/dx | dy/dx | dy/dx |
| **Easy** |  |  |  |  |  |
| Man w/o minor co-resident children (vs woman w/o minor co-resident children) | 0.1650** | 0.0188 | 0.1860** | -0.0344 | -0.0021 |
|  | (0.0706) | (0.0681) | (0.0756) | (0.0776) | (0.0763) |
| Father (vs Mother) | 0.1080** | 0.0146 | 0.0452 | -0.0231 | 0.0563 |
|  | (0.0472) | (0.0453) | (0.0491) | (0.0481) | (0.0461) |
| **Neutral** |  |  |  |  |  |
| Man w/o minor co-resident children (vs woman w/o minor co-resident children) | -0.1630** | 0.0237 | -0.177** | 0.0356 | -0.0304 |
|  | (0.0669) | (0.0672) | (0.0724) | (0.0727) | (0.0706) |
| Father (vs Mother) | -0.0779* | -0.0361 | -0.0016 | 0.0420 | -0.0056 |
|  | (0.0437) | (0.0426) | (0.0473) | (0.0442) | (0.0430) |
| **Difficult** |  |  |  |  |  |
| Man w/o minor co-resident children (vs woman w/o minor co-resident children) | -0.0014 | -0.0425 | -0.0090 | -0.0011 | 0.0325 |
|  | (0.0571) | (0.0464) | (0.0544) | (0.0553) | (0.0608) |
| Father (vs Mother) | -0.0300 | 0.0215 | -0.0436* | -0.0189 | -0.0507* |
|  | (0.0380) | (0.0290) | (0.0249) | (0.0330) | (0.0277) |
| Observations | 700 | 737 | 633 | 634 | 628 |

Note: Standard errors in parentheses. *** p<0.01, ** p<0.05, * p<0.1. Controlled for all co-variates. Reference categories are women, non-essential occupations, partner in non-essential occupation, vocational education, no minor co-resident children, neutral on statement ‘I can decide where I work’, partner working on location due to the nature of the work.
