# Supplementary material for: Gender inequality in work location, childcare and work-life balance: Phase-specific differences throughout the COVID-19 pandemic
Source: PLoS One. 2024 Jun 25;19(6):e0302633. doi: 10.1371/journal.pone.0302633 (PMC11198899; doi:10.1371/journal.pone.0302633)
Supplement: S12 Table — Note: Standard errors in parentheses. *** p<0.01, ** p<0.05, * p<0.1. Controlled for all co-variates. Reference categories are women, non-essential occupations, partner in non-essential occupation, vocational education, no minor co-resident children, neutral on statement ‘I can decide where I work’, partner working on location due to the nature of the work. (DOCX) [file pone.0302633.s013.docx]

**S12 Table. Marginal effect of gender on work-life balance in essential and non-essential occupations.**

|  | Apr-20 | Jun-20 | Sept-20 | Nov-20 | Nov 21 |
| --- | --- | --- | --- | --- | --- |
|  | dy/dx | dy/dx | dy/dx | dy/dx | dy/dx |
| **Easy** |  |  |  |  |  |
| Man non-essential (vs woman non-essential) | 0.0286 | 0.102* | 0.0373 | 0.0722 | -0.0297 |
|  | (0.0540) | (0.0527) | (0.0504) | (0.0579) | (0.0557) |
| Man essential (vs woman essential) | -0.0714 | 0.1530*** | -0.00562 | 0.0993* | -0.0220 |
|  | (0.0579) | (0.0593) | (0.0573) | (0.0594) | (0.0608) |
| **Neutral** |  |  |  |  |  |
| Man non-essential (vs woman non-essential) | 0.0227 | -0.0519 | 0.0208 | -0.0319 | 0.0482 |
|  | (0.0524) | (0.0491) | (0.0481) | (0.0553) | (0.0504) |
| Man essential (vs woman essential) | 0.0472 | -0.1670*** | -0.0642 | -0.0715 | 0.0316 |
|  | (0.0587) | (0.0540) | (0.0537) | (0.0577) | (0.0569) |
| **Difficult** |  |  |  |  |  |
| Man non-essential (vs woman non-essential) | 0.0242 | 0.0140 | 0.0698* | -0.0278 | -0.0096 |
|  | (0.0541) | (0.0504) | (0.0403) | (0.0329) | (0.0405) |
| Man essential (vs woman essential) | -0.0513 | -0.0499 | -0.0581* | -0.0403 | -0.0185 |
|  | (0.0490) | (0.0414) | (0.0335) | (0.0345) | (0.0399) |
| Observations | 641 | 700 | 737 | 633 | 634 |

Note: Standard errors in parentheses. *** p<0.01, ** p<0.05, * p<0.1. Controlled for all co-variates. Reference categories are women, non-essential occupations, partner in non-essential occupation, vocational education, no minor co-resident children, neutral on statement ‘I can decide where I work’, partner working on location due to the nature of the work.
