# Supplementary material for: Gender inequality in work location, childcare and work-life balance: Phase-specific differences throughout the COVID-19 pandemic
Source: PLoS One. 2024 Jun 25;19(6):e0302633. doi: 10.1371/journal.pone.0302633 (PMC11198899; doi:10.1371/journal.pone.0302633)
Supplement: S13 Table — Note: Standard errors in parentheses. *** p<0.01, ** p<0.05, * p<0.1. Controlled for all co-variates. Reference categories are women, non-essential occupations, partner in non-essential occupation, vocational education, no minor co-resident children, neutral on statement ‘I can decide where I work’, partner working on location due to the nature of the work. (DOCX) [file pone.0302633.s014.docx]

**S13 Table. Marginal effect of gender on work-life balance across educational groups.**

|  | Apr-20 | Jun-20 | Sept-20 | Nov-20 | Nov 21 | Apr-22 |
| --- | --- | --- | --- | --- | --- | --- |
|  | dy/dx | dy/dx | dy/dx | dy/dx | dy/dx | dy/dx |
| **Easy** |  |  |  |  |  |  |
| Prim. / sec. educated man (vs prim. / sec. educated woman) | 0.1160 | 0.1090 | 0.1550 | 0.00321 | 0.0141 | -0.0836 |
|  | (0.1290) | (0.1110) | (0.1020) | (0.1170) | (0.1170) | (0.1130) |
| Vocational educated man (vs vocational educated woman) | -0.1150 | 0.0324 | 0.00461 | 0.0398 | -0.1220* | 0.0594 |
|  | (0.0707) | (0.0723) | (0.0699) | (0.0747) | (0.0733) | (0.0737) |
| Tertiary educated man (vs tertiary educated woman) | 0.0144 | 0.1700*** | -0.00569 | 0.1230** | 0.0137 | 0.0555 |
|  | (0.0506) | (0.0499) | (0.0487) | (0.0530) | (0.0531) | (0.0512) |
| **Neutral** |  |  |  |  |  |  |
| Prim. / sec. educated man (vs prim. / sec. educated woman) | -0.0259 | -0.1130 | -0.1630* | 0.00344 | 0.0470 | 0.0358 |
|  | (0.1290) | (0.1070) | (0.0983) | (0.116) | (0.1120) | (0.1020) |
| Vocational educated man (vs vocational educated woman) | 0.0428 | -0.0317 | -0.0249 | 0.0145 | 0.1380** | 0.0107 |
|  | (0.0707) | (0.0702) | (0.0688) | (0.0721) | (0.0689) | (0.0706) |
| Tertiary educated man (vs tertiary educated woman) | 0.0409 | -0.1340*** | 0.0144 | -0.0936* | -0.0106 | -0.0334 |
|  | (0.0491) | (0.0454) | (0.0458) | (0.0505) | (0.0482) | (0.0473) |
| **Difficult** |  |  |  |  |  |  |
| Prim. / sec. educated man (vs prim. / sec. educated woman) | -0.0899 | 0.00439 | 0.00879 | -0.00665 | -0.0611 | 0.0478 |
|  | (0.0825) | (0.0750) | (0.0561) | (0.0449) | (0.0781) | (0.0782) |
| Vocational educated man (vs vocational educated woman) | 0.0725 | -0.0007 | 0.0203 | -0.0544 | -0.0169 | -0.0701 |
|  | (0.0596) | (0.0551) | (0.0399) | (0.0453) | (0.0468) | (0.0457) |
| Tertiary educated man (vs tertiary educated woman) | -0.0553 | -0.0353 | -0.00873 | -0.0296 | -0.00305 | -0.0221 |
|  | (0.0498) | (0.0426) | (0.0347) | (0.0317) | (0.0385) | (0.0353) |
| Observations | 641 | 700 | 737 | 633 | 634 | 628 |

Note: Standard errors in parentheses. *** p<0.01, ** p<0.05, * p<0.1. Controlled for all co-variates. Reference categories are women, non-essential occupations, partner in non-essential occupation, vocational education, no minor co-resident children, neutral on statement ‘I can decide where I work’, partner working on location due to the nature of the work.
