# Supplementary material for: Gender inequality in work location, childcare and work-life balance: Phase-specific differences throughout the COVID-19 pandemic
Source: PLoS One. 2024 Jun 25;19(6):e0302633. doi: 10.1371/journal.pone.0302633 (PMC11198899; doi:10.1371/journal.pone.0302633)
Supplement: S14 Table — Note: *** p<0.01, ** p<0.05, * p<0.1. Reference categories are women, non-essential occupations, partner in non-essential occupation, vocational education, partner working on location due to the nature of the work. (DOCX) [file pone.0302633.s015.docx]

**S14 Table. Multinomial logits of work location, including estimated average marginal effects of all covariates in April 2020.**

| April 2020 (n=617) | **Fully from home** | | **Partially from home** | | **Workplace – can work from home** | | **Workplace - nature of the work** | |
| --- | --- | --- | --- | --- | --- | --- | --- | --- |
|  | dy/dx | S.E. | dy/dx | S.E. | dy/dx | S.E. | dy/dx | S.E. |
| Men | -0.0700* | (0.0379) | 0.0549** | (0.0280) | 0.0407* | (0.0228) | -0.0256 | (0.0355) |
| Essential occupation | -0.268*** | (0.0333) | 0.0541** | (0.0257) | 0.0493** | (0.0213) | 0.165*** | (0.0328) |
| Partner in essential occupation | 0.0850** | (0.0402) | -0.0265 | (0.0302) | -0.00541 | (0.0232) | -0.0531 | (0.0380) |
| Age | -0.00272 | (0.00245) | -0.00137 | (0.00180) | 0.00285* | (0.00147) | 0.00124 | (0.00234) |
| Prim. / sec. education | -0.0184 | (0.0806) | -0.0444 | (0.0597) | -0.0228 | (0.0510) | 0.0857 | (0.0556) |
| Tertiary education | 0.284*** | (0.0355) | -0.0212 | (0.0262) | 0.0245 | (0.0222) | -0.287*** | (0.0276) |
| Partner working fully from home | 0.170*** | (0.0445) | 0.0592* | (0.0357) | -0.0163 | (0.0265) | -0.213*** | (0.0384) |
| Partner working hybrid | 0.107* | (0.0574) | 0.0931** | (0.0416) | -0.00271 | (0.0334) | -0.197*** | (0.0534) |
| Partner working on location,  possibility to work from home | 0.0205 | (0.0826) | 0.0184 | (0.0680) | 0.0532 | (0.0370) | -0.0921 | (0.0703) |
| Partner not working | 0.0904 | (0.0642) | 0.0317 | (0.0487) | -0.00542 | (0.0362) | -0.117** | (0.0551) |

Note: *** p<0.01, ** p<0.05, * p<0.1. Reference categories are women, non-essential occupations, partner in non-essential occupation, vocational education, partner working on location due to the nature of the work.
