# Supplementary material for: Gender inequality in work location, childcare and work-life balance: Phase-specific differences throughout the COVID-19 pandemic
Source: PLoS One. 2024 Jun 25;19(6):e0302633. doi: 10.1371/journal.pone.0302633 (PMC11198899; doi:10.1371/journal.pone.0302633)
Supplement: S15 Table — Note: *** p<0.01, ** p<0.05, * p<0.1. Reference categories are women, non-essential occupations, partner in non-essential occupation, vocational education, no minor co-resident children, neutral on statement ‘I can decide where I work’, partner working on location due to the nature of the work. (DOCX) [file pone.0302633.s016.docx]

**S15 Table. Multinomial logits of work location, including estimated average marginal effects of all covariates in June 2020.**

| June 2020 (n=764) | **Fully from home** | | **Partially from home** | | **Workplace – can work from home** | | **Workplace - nature of the work** | |
| --- | --- | --- | --- | --- | --- | --- | --- | --- |
|  | dy/dx | S.E. | dy/dx | S.E. | dy/dx | S.E. | dy/dx | S.E. |
| Men | -0.0447 | (0.0318) | 0.0616** | (0.0269) | 0.0131 | (0.0226) | -0.0301 | (0.0299) |
| Essential occupation | -0.302*** | (0.0312) | 0.0663** | (0.0281) | 0.0488** | (0.0241) | 0.187*** | (0.0302) |
| Partner in essential occupation | 0.0356 | (0.0372) | -0.0342 | (0.0284) | 0.0188 | (0.0258) | -0.0202 | (0.0329) |
| Age | 0.00135 | (0.00191) | -0.000941 | (0.00162) | -0.00115 | (0.00136) | 0.000744 | (0.00180) |
| Prim. / sec. education | -0.00712 | (0.0519) | 0.000356 | (0.0475) | 0.0173 | (0.0482) | -0.0106 | (0.0534) |
| Tertiary education | 0.199*** | (0.0364) | 0.0704** | (0.0297) | -0.0186 | (0.0271) | -0.251*** | (0.0356) |
| Co-resident minor child | -0.0451 | (0.0325) | 0.0168 | (0.0267) | 0.0229 | (0.0216) | 0.00540 | (0.0295) |
| Workplace autonomy - disagree | -0.0847 | (0.0827) | -0.0759 | (0.0726) | -0.0690 | (0.0654) | 0.230*** | (0.0864) |
| Workplace autonomy - agree | 0.00801 | (0.0843) | 0.136* | (0.0764) | 0.0540 | (0.0689) | -0.198** | (0.0858) |
| Workplace autonomy - NA | -0.0983 | (0.0965) | -0.122 | (0.0769) | -0.108 | (0.0672) | 0.328*** | (0.0997) |
| Partner working fully from home | 0.128*** | (0.0402) | -0.0264 | (0.0345) | 0.00282 | (0.0281) | -0.105*** | (0.0376) |
| Partner working hybrid | 0.101* | (0.0521) | 0.0191 | (0.0455) | -0.0288 | (0.0306) | -0.0908* | (0.0474) |
| Partner working on location,  possibility to work from home | 0.0585 | (0.0582) | -0.0701 | (0.0470) | 0.0451 | (0.0464) | -0.0334 | (0.0568) |
| Partner not working | 0.179*** | (0.0531) | -0.0756* | (0.0402) | 0.0114 | (0.0388) | -0.115** | (0.0479) |

Note: *** p<0.01, ** p<0.05, * p<0.1. Reference categories are women, non-essential occupations, partner in non-essential occupation, vocational education, no minor co-resident children, neutral on statement ‘I can decide where I work’, partner working on location due to the nature of the work.
