# Supplementary material for: Gender inequality in work location, childcare and work-life balance: Phase-specific differences throughout the COVID-19 pandemic
Source: PLoS One. 2024 Jun 25;19(6):e0302633. doi: 10.1371/journal.pone.0302633 (PMC11198899; doi:10.1371/journal.pone.0302633)
Supplement: S16 Table — Note: *** p<0.01, ** p<0.05, * p<0.1. Reference categories are women, non-essential occupations, partner in non-essential occupation, vocational education, no minor co-resident children, neutral on statement ‘I can decide where I work’, partner working on location due to the nature of the work. (DOCX) [file pone.0302633.s017.docx]

**S16 Table. Multinomial logits of work location, including estimated average marginal effects of all covariates in September 2020.**

| September 2020 (n=798) | **Fully from home** | | **Partially from home** | | **Workplace – can work from home** | | **Workplace - nature of the work** | |
| --- | --- | --- | --- | --- | --- | --- | --- | --- |
|  | dy/dx | S.E. | dy/dx | S.E. | dy/dx | S.E. | dy/dx | S.E. |
| Men | -0.0231 | (0.0299) | 0.00503 | (0.0251) | 0.0553** | (0.0270) | -0.0372 | (0.0285) |
| Essential occupation | -0.192*** | (0.0300) | -0.0354 | (0.0261) | 0.0265 | (0.0290) | 0.201*** | (0.0302) |
| Partner in essential occupation | 0.0788** | (0.0376) | 0.00427 | (0.0302) | 0.00457 | (0.0308) | -0.0876*** | (0.0318) |
| Age | 0.000556 | (0.00182) | 0.00112 | (0.00151) | -0.000460 | (0.00163) | -0.00121 | (0.00175) |
| Prim. / sec. education | 0.0112 | (0.0473) | 0.0381 | (0.0441) | -0.0773* | (0.0431) | 0.0280 | (0.0463) |
| Tertiary education | 0.130*** | (0.0342) | 0.0689** | (0.0286) | -0.0134 | (0.0331) | -0.186*** | (0.0335) |
| Co-resident minor child | -0.0209 | (0.0306) | 0.00358 | (0.0253) | 0.00774 | (0.0271) | 0.00957 | (0.0288) |
| Workplace autonomy - disagree | 0.00267 | (0.0717) | -0.0846 | (0.0625) | -0.205** | (0.0817) | 0.287*** | (0.0761) |
| Workplace autonomy - agree | 0.0902 | (0.0740) | 0.168** | (0.0677) | -0.0751 | (0.0847) | -0.183** | (0.0761) |
| Workplace autonomy - NA | -0.0511 | (0.0906) | -0.149** | (0.0612) | -0.262*** | (0.0879) | 0.462*** | (0.0951) |
| Partner working fully from home | 0.116*** | (0.0411) | -0.00601 | (0.0353) | -0.0105 | (0.0353) | -0.0992*** | (0.0372) |
| Partner working hybrid | 0.0513 | (0.0444) | -0.0119 | (0.0383) | 0.0474 | (0.0431) | -0.0868** | (0.0430) |
| Partner working on location,  possibility to work from home | 0.0525 | (0.0466) | -0.0649* | (0.0372) | 0.0632 | (0.0464) | -0.0508 | (0.0457) |
| Partner not working | 0.114** | (0.0524) | -0.0135 | (0.0424) | -0.0181 | (0.0418) | -0.0824* | (0.0470) |

Note: *** p<0.01, ** p<0.05, * p<0.1. Reference categories are women, non-essential occupations, partner in non-essential occupation, vocational education, no minor co-resident children, neutral on statement ‘I can decide where I work’, partner working on location due to the nature of the work.
