# Supplementary material for: Gender inequality in work location, childcare and work-life balance: Phase-specific differences throughout the COVID-19 pandemic
Source: PLoS One. 2024 Jun 25;19(6):e0302633. doi: 10.1371/journal.pone.0302633 (PMC11198899; doi:10.1371/journal.pone.0302633)
Supplement: S17 Table — Note: *** p<0.01, ** p<0.05, * p<0.1. Reference categories are women, non-essential occupations, partner in non-essential occupation, vocational education, no minor co-resident children, neutral on statement ‘I can decide where I work’, partner working on location due to the nature of the work. (DOCX) [file pone.0302633.s018.docx]

**S17 Table. Multinomial logits of work location, including estimated average marginal effects of all covariates in November 2020.**

| November 2020 (n=702) | **Fully from home** | | **Partially from home** | | **Workplace – can work from home** | | **Workplace - nature of the work** | |
| --- | --- | --- | --- | --- | --- | --- | --- | --- |
|  | dy/dx | S.E. | dy/dx | S.E. | dy/dx | S.E. | dy/dx | S.E. |
| Men | -0.0561* | (0.0336) | 0.00305 | (0.0246) | 0.0299 | (0.0260) | 0.0231 | (0.0330) |
| Essential occupation | -0.304*** | (0.0329) | 0.0646** | (0.0256) | 0.0189 | (0.0264) | 0.221*** | (0.0336) |
| Partner in essential occupation | 0.0561 | (0.0379) | -0.00253 | (0.0271) | -0.0169 | (0.0273) | -0.0367 | (0.0356) |
| Age | 0.00121 | (0.00202) | -0.00102 | (0.00148) | 0.000549 | (0.00158) | -0.000737 | (0.00201) |
| Prim. / sec. education | -0.0792* | (0.0450) | 0.0285 | (0.0445) | -0.00263 | (0.0399) | 0.0534 | (0.0531) |
| Tertiary education | 0.188*** | (0.0380) | 0.0106 | (0.0283) | 0.0253 | (0.0285) | -0.224*** | (0.0384) |
| Co-resident minor child | -0.0629* | (0.0345) | -0.0160 | (0.0254) | 0.0275 | (0.0250) | 0.0513 | (0.0325) |
| Workplace autonomy - disagree | 0.0118 | (0.0778) | -0.138* | (0.0771) | -0.0103 | (0.0621) | 0.137 | (0.0916) |
| Workplace autonomy - agree | 0.0722 | (0.0805) | 0.115 | (0.0826) | 0.0854 | (0.0662) | -0.272*** | (0.0921) |
| Workplace autonomy - NA | 0.0388 | (0.0987) | -0.196** | (0.0763) | -0.0891 | (0.0639) | 0.246** | (0.110) |
| Partner working fully from home | 0.0870** | (0.0420) | 0.0683** | (0.0324) | -0.00763 | (0.0326) | -0.148*** | (0.0405) |
| Partner working hybrid | 0.0287 | (0.0545) | 0.0797* | (0.0446) | -0.0460 | (0.0377) | -0.0624 | (0.0545) |
| Partner working on location,  possibility to work from home | -0.00687 | (0.0579) | -0.0111 | (0.0404) | 0.0889 | (0.0569) | -0.0709 | (0.0587) |
| Partner not working | 0.144** | (0.0560) | -0.0209 | (0.0325) | -0.0418 | (0.0355) | -0.0809 | (0.0537) |

Note: *** p<0.01, ** p<0.05, * p<0.1. Reference categories are women, non-essential occupations, partner in non-essential occupation, vocational education, no minor co-resident children, neutral on statement ‘I can decide where I work’, partner working on location due to the nature of the work.
