# Supplementary material for: Gender inequality in work location, childcare and work-life balance: Phase-specific differences throughout the COVID-19 pandemic
Source: PLoS One. 2024 Jun 25;19(6):e0302633. doi: 10.1371/journal.pone.0302633 (PMC11198899; doi:10.1371/journal.pone.0302633)
Supplement: S18 Table — Note: *** p<0.01, ** p<0.05, * p<0.1. Reference categories are women, non-essential occupations, partner in non-essential occupation, vocational education, no minor co-resident children, neutral on statement ‘I can decide where I work’, partner working on location due to the nature of the work. (DOCX) [file pone.0302633.s019.docx]

**S18 Table. Multinomial logits of work location, including estimated average marginal effects of all covariates in November 2021.**

| November 2021 (n=709) | **Fully from home** | | **Partially from home** | | **Workplace – can work from home** | | **Workplace - nature of the work** | |
| --- | --- | --- | --- | --- | --- | --- | --- | --- |
|  | dy/dx | S.E. | dy/dx | S.E. | dy/dx | S.E. | dy/dx | S.E. |
| Men | -0.0511* | (0.0292) | 0.00431 | (0.0276) | 0.000942 | (0.0251) | 0.0459* | (0.0275) |
| Essential occupation | -0.141*** | (0.0291) | 0.0190 | (0.0289) | -0.000691 | (0.0270) | 0.123*** | (0.0299) |
| Partner in essential occupation | -0.000348 | (0.0336) | -0.0329 | (0.0298) | 0.0543* | (0.0308) | -0.0210 | (0.0313) |
| Age | -0.00205 | (0.00177) | 0.00216 | (0.00167) | -0.00133 | (0.00159) | 0.00123 | (0.00177) |
| Prim. / sec. education | -0.0234 | (0.0451) | -0.0910** | (0.0437) | 0.0359 | (0.0520) | 0.0785* | (0.0469) |
| Tertiary education | 0.0797** | (0.0338) | 0.0363 | (0.0335) | -0.00391 | (0.0299) | -0.112*** | (0.0315) |
| Co-resident minor child | -0.0279 | (0.0306) | 0.00113 | (0.0285) | 0.00317 | (0.0261) | 0.0236 | (0.0290) |
| Workplace autonomy - disagree | -0.0566 | (0.0759) | -0.0683 | (0.0731) | -0.165* | (0.0973) | 0.290*** | (0.102) |
| Workplace autonomy - agree | 0.124 | (0.0795) | 0.296*** | (0.0795) | -0.0928 | (0.0993) | -0.327*** | (0.101) |
| Workplace autonomy - NA | -0.0756 | (0.0845) | -0.115 | (0.0739) | -0.254*** | (0.0971) | 0.445*** | (0.109) |
| Partner working fully from home | 0.0538 | (0.0435) | 0.0171 | (0.0378) | 0.0623 | (0.0393) | -0.133*** | (0.0419) |
| Partner working hybrid | -0.0713** | (0.0342) | 0.0648* | (0.0363) | 0.0463 | (0.0348) | -0.0398 | (0.0376) |
| Partner working on location,  possibility to work from home | -0.00341 | (0.0502) | 0.0954* | (0.0515) | -0.00370 | (0.0408) | -0.0883* | (0.0498) |
| Partner not working | 0.0910* | (0.0537) | 0.00634 | (0.0435) | 0.0258 | (0.0431) | -0.123** | (0.0479) |

Note: *** p<0.01, ** p<0.05, * p<0.1. Reference categories are women, non-essential occupations, partner in non-essential occupation, vocational education, no minor co-resident children, neutral on statement ‘I can decide where I work’, partner working on location due to the nature of the work.
