# Supplementary material for: Gender inequality in work location, childcare and work-life balance: Phase-specific differences throughout the COVID-19 pandemic
Source: PLoS One. 2024 Jun 25;19(6):e0302633. doi: 10.1371/journal.pone.0302633 (PMC11198899; doi:10.1371/journal.pone.0302633)
Supplement: S19 Table — Note: *** p<0.01, ** p<0.05, * p<0.1. Reference categories are women, non-essential occupations, partner in non-essential occupation, vocational education, no minor co-resident children, neutral on statement ‘I can decide where I work’, partner working on location due to the nature of the work. (DOCX) [file pone.0302633.s020.docx]

**S19 Table. Multinomial logits of work location, including estimated average marginal effects of all covariates in April 2022.**

| April 2022 (n=681) | **Fully from home** | | **Partially from home** | | **Workplace – can work from home** | | **Workplace - nature of the work** | |
| --- | --- | --- | --- | --- | --- | --- | --- | --- |
|  | dy/dx | S.E. | dy/dx | S.E. | dy/dx | S.E. | dy/dx | S.E. |
| Men | -0.0184 | (0.0252) | -0.000717 | (0.0292) | 0.0142 | (0.0291) | 0.00490 | (0.0287) |
| Age | 0.00232 | (0.00160) | 0.000409 | (0.00182) | 0.000342 | (0.00184) | -0.00307* | (0.00183) |
| Prim. / sec. education | -0.0349 | (0.0470) | 0.0427 | (0.0535) | -0.0359 | (0.0551) | 0.0281 | (0.0506) |
| Tertiary education | 0.00005 | (0.0328) | 0.165*** | (0.0325) | -0.0168 | (0.0362) | -0.148*** | (0.0348) |
| Co-resident minor child | -0.0226 | (0.0278) | 0.0228 | (0.0310) | 0.00947 | (0.0310) | -0.00963 | (0.0305) |
| Workplace autonomy - disagree | -0.0768 | (0.0631) | -0.392*** | (0.0964) | 0.0106 | (0.0756) | 0.459*** | (0.0785) |
| Workplace autonomy - agree | 0.150** | (0.0690) | -0.0874 | (0.0997) | 0.0594 | (0.0774) | -0.122 | (0.0763) |
| Workplace autonomy - NA | -0.0153 | (0.0701) | -0.498*** | (0.0946) | -0.161** | (0.0730) | 0.675*** | (0.0813) |
| Partner working fully from home | 0.0882** | (0.0439) | 0.0280 | (0.0452) | -0.0178 | (0.0443) | -0.0984** | (0.0469) |
| Partner working hybrid | -0.00220 | (0.0311) | 0.0619 | (0.0383) | 0.0202 | (0.0397) | -0.0799** | (0.0380) |
| Partner working on location,  possibility to work from home | 0.0214 | (0.0443) | -0.00422 | (0.0478) | 0.0367 | (0.0535) | -0.0539 | (0.0518) |
| Partner not working | 0.0327 | (0.0416) | 0.0364 | (0.0475) | -0.0466 | (0.0426) | -0.0225 | (0.0450) |

Note: *** p<0.01, ** p<0.05, * p<0.1. Reference categories are women, non-essential occupations, partner in non-essential occupation, vocational education, no minor co-resident children, neutral on statement ‘I can decide where I work’, partner working on location due to the nature of the work.
