# Supplementary material for: Gender inequality in work location, childcare and work-life balance: Phase-specific differences throughout the COVID-19 pandemic
Source: PLoS One. 2024 Jun 25;19(6):e0302633. doi: 10.1371/journal.pone.0302633 (PMC11198899; doi:10.1371/journal.pone.0302633)
Supplement: S20 Table — Note: *** p<0.01, ** p<0.05, * p<0.1. Reference categories are mothers, non-essential occupations, partner in non-essential occupation, vocational education, partner working on location due to the nature of the work. (DOCX) [file pone.0302633.s021.docx]

**S20 Table. Multinomial logits of division of childcare, including estimated average marginal effects of all covariates in April 2020.**

| April 2020 (n=603) | **More childcare** | | **Same amount of childcare** | | **Less childcare** | |
| --- | --- | --- | --- | --- | --- | --- |
|  | dy/dx | S.E. | dy/dx | S.E. | dy/dx | S.E. |
| Fathers | 0.0866*** | (0.0328) | -0.0303 | (0.0436) | -0.0562 | (0.0342) |
| Essential occupation | -0.1217*** | (0.0326) | -0.0006 | (0.0419) | 0.1223*** | (0.0311) |
| Partner in essential occupation | 0.0108 | (0.0342) | 0.0358 | (0.0448) | -0.0467 | (0.0355) |
| Age | 0.0009 | (0.0034) | 0.0042 | (0.0045) | -0.0051 | (0.0036) |
| Prim. / sec. education | -0.1346* | (0.0817) | 0.0555 | (0.0851) | 0.0790 | (0.0541) |
| Tertiary education | 0.0479 | (0.0343) | -0.0280 | (0.0449) | -0.0200 | (0.0359) |
| Partner working fully from home | -0.0609* | (0.0360) | -0.0295 | (0.0488) | 0.0904** | (0.0395) |
| Partner working hybrid | -0.1635*** | (0.0568) | 0.0821 | (0.0684) | 0.0814 | (0.0512) |
| Partner working on location,  possibility to work from home | -0.0720 | (0.0688) | -0.0673 | (0.0875) | 0.1394** | (0.0658) |
| Partner not working | -0.0689 | (0.0511) | 0.0752 | (0.0717) | -0.0063 | (0.0617) |
| Age youngest child | -0.0075 | (0.0046) | 0.0103* | (0.0059) | -0.0028 | (0.0047) |

Note: *** p<0.01, ** p<0.05, * p<0.1. Reference categories are mothers, non-essential occupations, partner in non-essential occupation, vocational education, partner working on location due to the nature of the work.
