# Supplementary material for: Gender inequality in work location, childcare and work-life balance: Phase-specific differences throughout the COVID-19 pandemic
Source: PLoS One. 2024 Jun 25;19(6):e0302633. doi: 10.1371/journal.pone.0302633 (PMC11198899; doi:10.1371/journal.pone.0302633)
Supplement: S21 Table — Note: *** p<0.01, ** p<0.05, * p<0.1. Reference categories are mothers, non-essential occupations, partner in non-essential occupation, vocational education, neutral on statement ‘I can decide where I work’, partner working on location due to the nature of the work. (DOCX) [file pone.0302633.s022.docx]

**S21 Table. Multinomial logits of division of childcare, including estimated average marginal effects of all covariates in June 2020.**

| June 2020 (n=522) | **More childcare** | | **Same amount of childcare** | | **Less childcare** | |
| --- | --- | --- | --- | --- | --- | --- |
|  | dy/dx | S.E. | dy/dx | S.E. | dy/dx | S.E. |
| Fathers | 0.0861** | (0.0410) | 0.0076 | (0.0487) | -0.0937** | (0.0451) |
| Essential occupation | -0.0211 | (0.0395) | -0.0017 | (0.0469) | 0.0229 | (0.0430) |
| Partner in essential occupation | 0.0392 | (0.0460) | -0.0701 | (0.0522) | 0.0309 | (0.0495) |
| Age | 0.0027 | (0.0041) | -0.0008 | (0.0050) | -0.0019 | (0.0045) |
| Prim. / sec. education | 0.0229 | (0.0670) | -0.1150 | (0.0759) | 0.0921 | (0.0777) |
| Tertiary education | -0.0046 | (0.0427) | 0.0815 | (0.0515) | -0.0768 | (0.0473) |
| Workplace autonomy - disagree | -0.0163 | (0.0917) | -0.0148 | (0.1140) | 0.0311 | (0.1017) |
| Workplace autonomy - agree | 0.0106 | (0.0941) | 0.0007 | (0.1170) | -0.0113 | (0.1041) |
| Workplace autonomy - NA | 0.0848 | (0.1060) | -0.1190 | (0.1266) | 0.0342 | (0.1140) |
| Partner working fully from home | -0.0658 | (0.0484) | 0.0126 | (0.0584) | 0.0532 | (0.0527) |
| Partner working hybrid | -0.0974* | (0.0537) | 0.0522 | (0.0694) | 0.0452 | (0.0632) |
| Partner working on location,  possibility to work from home | 0.0371 | (0.0765) | -0.0940 | (0.0838) | 0.0569 | (0.0781) |
| Partner not working | 0.0418 | (0.0656) | -0.0874 | (0.0711) | 0.0456 | (0.0672) |
| Age youngest child | -0.0032 | (0.0055) | 0.0018 | (0.0067 | 0.0014 | (0.0060) |

Note: *** p<0.01, ** p<0.05, * p<0.1. Reference categories are mothers, non-essential occupations, partner in non-essential occupation, vocational education, neutral on statement ‘I can decide where I work’, partner working on location due to the nature of the work.
