# Supplementary material for: Gender inequality in work location, childcare and work-life balance: Phase-specific differences throughout the COVID-19 pandemic
Source: PLoS One. 2024 Jun 25;19(6):e0302633. doi: 10.1371/journal.pone.0302633 (PMC11198899; doi:10.1371/journal.pone.0302633)
Supplement: S22 Table — Note: *** p<0.01, ** p<0.05, * p<0.1. Reference categories are mothers, non-essential occupations, partner in non-essential occupation, vocational education, neutral on statement ‘I can decide where I work’, partner working on location due to the nature of the work. (DOCX) [file pone.0302633.s023.docx]

**S22 Table. Multinomial logits of division of childcare, including estimated average marginal effects of all covariates in September 2020.**

| September 2020 (n=543) | **More childcare** | | **Same amount of childcare** | | **Less childcare** | |
| --- | --- | --- | --- | --- | --- | --- |
|  | dy/dx | S.E. | dy/dx | S.E. | dy/dx | S.E. |
| Fathers | 0.0817** | (0.0366) | 0.0971** | (0.0480) | -0.1788*** | (0.0437) |
| Essential occupation | -0.0214 | (0.0352) | 0.0479 | (0.0461) | -0.0265 | (0.0414) |
| Partner in essential occupation | -0.0214 | (0.0387) | -0.0008 | (0.0522) | 0.0221 | (0.0488) |
| Age | -0.0042 | (0.0037) | 0.0073 | (0.0049) | -0.0031 | (0.0045) |
| Prim. / sec. education | -0.0238 | (0.0575) | -0.0869 | (0.0748) | 0.1108 | (0.0738) |
| Tertiary education | -0.0383 | (0.0399) | 0.0802 | (0.0512) | -0.0420 | (0.0464) |
| Workplace autonomy - disagree | -0.1142 | (0.0841) | 0.1008 | (0.1019) | 0.0134 | (0.0934) |
| Workplace autonomy - agree | -0.0128 | (0.0880) | 0.0170 | (0.1049) | -0.0042 | (0.0960) |
| Workplace autonomy - NA | -0.0692 | (0.0950) | 0.0344 | (0.1174) | 0.0347 | (0.1069) |
| Partner working fully from home | -0.1071** | (0.0414) | -0.0031 | (0.0600) | 0.1103** | (0.0554) |
| Partner working hybrid | -0.0035 | (0.0530) | -0.0461 | (0.0651) | 0.0496 | (0.0583) |
| Partner working on location,  possibility to work from home | 0.0038 | (0.0595) | -0.0282 | (0.0724) | 0.0244 | (0.0632) |
| Partner not working | -0.0229 | (0.0547) | -0.0542 | (0.0738) | 0.0771 | (0.0700) |
| Age youngest child | 0.0066 | (0.0049) | -0.0039 | (0.0065) | -0.0027 | (0.0059) |

Note: *** p<0.01, ** p<0.05, * p<0.1. Reference categories are mothers, non-essential occupations, partner in non-essential occupation, vocational education, neutral on statement ‘I can decide where I work’, partner working on location due to the nature of the work.
