# Supplementary material for: Gender inequality in work location, childcare and work-life balance: Phase-specific differences throughout the COVID-19 pandemic
Source: PLoS One. 2024 Jun 25;19(6):e0302633. doi: 10.1371/journal.pone.0302633 (PMC11198899; doi:10.1371/journal.pone.0302633)
Supplement: S23 Table — Note: *** p<0.01, ** p<0.05, * p<0.1. Reference categories are mothers, non-essential occupations, partner in non-essential occupation, vocational education, neutral on statement ‘I can decide where I work’, partner working on location due to the nature of the work. (DOCX) [file pone.0302633.s024.docx]

**S23 Table. Multinomial logits of division of childcare, including estimated average marginal effects of all covariates in November 2020.**

| November 2020 (n=480) | **More childcare** | | **Same amount of childcare** | | **Less childcare** | |
| --- | --- | --- | --- | --- | --- | --- |
|  | dy/dx | S.E. | dy/dx | S.E. | dy/dx | S.E. |
| Fathers | -0.0134 | (0.0400) | 0.1520*** | (0.0492) | -0.1386*** | (0.0451) |
| Essential occupation | 0.0191 | (0.0389) | -0.0210 | (0.0482) | 0.0019 | (0.0434) |
| Partner in essential occupation | 0.0253 | (0.0432) | -0.0108 | (0.0520) | -0.0145 | (0.0477) |
| Age | 0.0006 | (0.0040) | 0.0002 | (0.0050) | -0.0008 | (0.0045) |
| Prim. / sec. education | -0.0731 | (0.0605) | -0.0124 | (0.0758) | 0.0854 | (0.0761) |
| Tertiary education | -0.0817* | (0.0442) | 0.2095*** | (0.0527) | -0.1278*** | (0.0490) |
| Workplace autonomy - disagree | 0.0588 | (0.0869) | -0.0959 | (0.1167) | 0.0371 | (0.105) |
| Workplace autonomy - agree | 0.0971 | (0.0909) | -0.1338 | (0.1201) | 0.0367 | (0.1087) |
| Workplace autonomy - NA | -0.0098 | (0.0949) | -0.0628 | (0.1339) | 0.0727 | (0.121) |
| Partner working fully from home | -0.0230 | (0.0462) | 0.0212 | (0.0588) | 0.0018 | (0.0528) |
| Partner working hybrid | -0.0817 | (0.0507) | 0.1058 | (0.0714) | -0.0241 | (0.0639) |
| Partner working on location,  possibility to work from home | 0.0655 | (0.0734) | -0.0787 | (0.0845) | 0.0132 | (0.0744) |
| Partner not working | 0.0448 | (0.0651) | -0.1425** | (0.0721) | 0.0977 | (0.0719) |
| Age youngest child | 0.0036 | (0.0052) | -0.0025 | (0.0065) | -0.0011 | (0.0059) |

Note: *** p<0.01, ** p<0.05, * p<0.1. Reference categories are mothers, non-essential occupations, partner in non-essential occupation, vocational education, neutral on statement ‘I can decide where I work’, partner working on location due to the nature of the work.
