# Supplementary material for: Gender inequality in work location, childcare and work-life balance: Phase-specific differences throughout the COVID-19 pandemic
Source: PLoS One. 2024 Jun 25;19(6):e0302633. doi: 10.1371/journal.pone.0302633 (PMC11198899; doi:10.1371/journal.pone.0302633)
Supplement: S24 Table — Note: *** p<0.01, ** p<0.05, * p<0.1. Reference categories are mothers, non-essential occupations, partner in non-essential occupation, vocational education, neutral on statement ‘I can decide where I work’, partner working on location due to the nature of the work. (DOCX) [file pone.0302633.s025.docx]

**S24 Table. Multinomial logits of division of childcare, including estimated average marginal effects of all covariates in November 2021.**

| November 2021 (n=479) | **More childcare** | | **Same amount of childcare** | | **Less childcare** | |
| --- | --- | --- | --- | --- | --- | --- |
|  | dy/dx | S.E. | dy/dx | S.E. | dy/dx | S.E. |
| Fathers | -0.0115 | (0.0415) | 0.0998** | (0.0505) | -0.0883* | (0.0478) |
| Essential occupation | 0.0026 | (0.0407) | -0.0238 | (0.0495) | 0.0212 | (0.0465) |
| Partner in essential occupation | -0.0034 | (0.0430) | 0.0422 | (0.0533) | -0.0388 | (0.0500) |
| Age | -0.0017 | (0.0044) | 0.0052 | (0.0054) | -0.0035 | (0.0051) |
| Prim. / sec. education | -0.0295 | (0.0629) | 0.0077 | (0.0812) | 0.0218 | (0.0774) |
| Tertiary education | 0.0005 | (0.0436) | 0.0518 | (0.0531) | -0.0523 | (0.0505) |
| Workplace autonomy - disagree | -0.2351* | (0.1244) | 0.1280 | (0.1171) | 0.1071 | (0.1078) |
| Workplace autonomy - agree | -0.1751 | (0.1273) | 0.1211 | (0.1200) | 0.0540 | (0.1100) |
| Workplace autonomy - NA | -0.3490*** | (0.1268) | 0.1297 | (0.1325) | 0.2192* | (0.1243) |
| Partner working fully from home | -0.0888 | (0.0554) | 0.1021 | (0.0689) | -0.0133 | (0.0623) |
| Partner working hybrid | -0.1470*** | (0.0474) | 0.1278** | (0.0627) | 0.0192 | (0.0586) |
| Partner working on location,  possibility to work from home | -0.0868 | (0.0693) | 0.0529 | (0.0887) | 0.0339 | (0.0835) |
| Partner not working | -0.0694 | (0.0624) | -0.0747 | (0.0729) | 0.1441* | (0.0749) |
| Age youngest child | 0.0082 | (0.0057) | -0.0123* | (0.0069) | 0.0041 | (0.0065) |

Note: *** p<0.01, ** p<0.05, * p<0.1. Reference categories are mothers, non-essential occupations, partner in non-essential occupation, vocational education, neutral on statement ‘I can decide where I work’, partner working on location due to the nature of the work.
