# Supplementary material for: Gender inequality in work location, childcare and work-life balance: Phase-specific differences throughout the COVID-19 pandemic
Source: PLoS One. 2024 Jun 25;19(6):e0302633. doi: 10.1371/journal.pone.0302633 (PMC11198899; doi:10.1371/journal.pone.0302633)
Supplement: S25 Table — Note: *** p<0.01, ** p<0.05, * p<0.1. Reference categories are mothers, non-essential occupations, partner in non-essential occupation, vocational education, neutral on statement ‘I can decide where I work’, partner working on location due to the nature of the work. (DOCX) [file pone.0302633.s026.docx]

**S25 Table.** **Multinomial logits of division of childcare, including estimated average marginal effects of all covariates in April 2022.**

| April 2022 (n=397) | **More childcare** | | **Same amount of childcare** | | **Less childcare** | |
| --- | --- | --- | --- | --- | --- | --- |
|  | dy/dx | S.E. | dy/dx | S.E. | dy/dx | S.E. |
| Fathers | 0.0988** | (0.0391) | 0.0177 | (0.0496) | -0.1165** | (0.0453) |
| Age | -0.0047 | (0.00422) | 0.0011 | (0.0054) | 0.0036 | (0.0050) |
| Prim. / sec. education | -0.0218 | (0.0632) | -0.0544 | (0.0818) | 0.0762 | (0.0799) |
| Tertiary education | -0.0235 | (0.0433) | 0.0883 | (0.0547) | -0.0648 | (0.0506) |
| Workplace autonomy - disagree | -0.0144 | (0.0946) | 0.1971 | (0.1247) | -0.1827 | (0.1280) |
| Workplace autonomy - agree | 0.0528 | (0.0968) | 0.1132 | (0.1258) | -0.1660 | (0.1291) |
| Workplace autonomy - NA | 0.0320 | (0.1036) | 0.0485 | (0.1345) | -0.0805 | (0.1372) |
| Partner working fully from home | -0.0716 | (0.0580) | -0.0201 | (0.0765) | 0.0917 | (0.0723) |
| Partner working hybrid | -0.0981** | (0.0465) | 0.0116 | (0.0643) | 0.0865 | (0.0605) |
| Partner working on location,  possibility to work from home | -0.0814 | (0.0624) | 0.0538 | (0.0819) | 0.0276 | (0.0742) |
| Partner not working | -0.0099 | (0.0599) | -0.0638 | (0.0743) | 0.0737 | (0.0701) |
| Age youngest child | 0.0033 | (0.0056) | -0.0049 | (0.0071) | 0.0016 | (0.0065) |

Note: *** p<0.01, ** p<0.05, * p<0.1. Reference categories are mothers, non-essential occupations, partner in non-essential occupation, vocational education, neutral on statement ‘I can decide where I work’, partner working on location due to the nature of the work.
