# Supplementary material for: Gender inequality in work location, childcare and work-life balance: Phase-specific differences throughout the COVID-19 pandemic
Source: PLoS One. 2024 Jun 25;19(6):e0302633. doi: 10.1371/journal.pone.0302633 (PMC11198899; doi:10.1371/journal.pone.0302633)
Supplement: S26 Table — Note: *** p<0.01, ** p<0.05, * p<0.1. Reference categories are women, non-essential occupations, partner in non-essential occupation, vocational education, partner working on location due to the nature of the work. (DOCX) [file pone.0302633.s027.docx]

**S26 Table. Multinomial logits of work-life balance, including estimated average marginal effects of all covariates in April 2020.**

| April 2020 (n=641) | **Easy** | | **Neutral** | | **Difficult** | |
| --- | --- | --- | --- | --- | --- | --- |
|  | dy/dx | S.E. | dy/dx | S.E. | dy/dx | S.E. |
| Men | -0.0163 | (0.0410) | 0.0339 | (0.0400) | -0.0176 | (0.0368) |
| Essential occupation | -0.0222 | (0.0397) | 0.0297 | (0.0388) | -0.0075 | (0.0360) |
| Partner in essential occupation | 0.0113 | (0.0444) | 0.0239 | (0.0431) | -0.0352 | (0.0388) |
| Age | 0.0099*** | (0.00261) | 0.0039 | (0.0026) | -0.0139*** | (0.0023) |
| Prim. / sec. education | 0.0855 | (0.0713) | 0.0641 | (0.0674) | -0.150* | (0.0867) |
| Tertiary education | -0.0488 | (0.0428) | -0.0898** | (0.0408) | 0.1390*** | (0.0392) |
| Workplace autonomy - disagree | 0.0710 | (0.0482) | -0.0623 | (0.0471) | -0.0087 | (0.0424) |
| Workplace autonomy - agree | -0.0359 | (0.0675) | 0.0031 | (0.0625) | 0.0329 | (0.0540) |
| Workplace autonomy - NA | 0.1450* | (0.0853) | 0.0339 | (0.0835) | -0.1790* | (0.0954) |
| Partner working fully from home | 0.0710 | (0.0482) | -0.0623 | (0.0471) | -0.0087 | (0.0424) |
| Partner working hybrid | -0.0359 | (0.0675) | 0.0031 | (0.0625) | 0.0329 | (0.0540) |
| Partner working on location,  possibility to work from home | 0.1450* | (0.0853) | 0.0339 | (0.0835) | -0.1790* | (0.0954) |
| Partner not working | 0.1640** | (0.0669) | 0.0636 | (0.0648) | -0.2280*** | (0.0716) |

Note: *** p<0.01, ** p<0.05, * p<0.1. Reference categories are women, non-essential occupations, partner in non-essential occupation, vocational education, partner working on location due to the nature of the work.
