# Supplementary material for: Gender inequality in work location, childcare and work-life balance: Phase-specific differences throughout the COVID-19 pandemic
Source: PLoS One. 2024 Jun 25;19(6):e0302633. doi: 10.1371/journal.pone.0302633 (PMC11198899; doi:10.1371/journal.pone.0302633)
Supplement: S27 Table — Note: *** p<0.01, ** p<0.05, * p<0.1. Reference categories are women, non-essential occupations, partner in non-essential occupation, vocational education, no minor co-resident children, neutral on statement ‘I can decide where I work’, partner working on location due to the nature of the work. (DOCX) [file pone.0302633.s028.docx]

**S27 Table. Multinomial logits of work-life balance, including estimated average marginal effects of all covariates in June 2020.**

| June 2020 (n=700) | **Easy** | | **Neutral** | | **Difficult** | |
| --- | --- | --- | --- | --- | --- | --- |
|  | dy/dx | S.E. | dy/dx | S.E. | dy/dx | S.E. |
| Men | 0.1240*** | (0.0403) | -0.1030*** | (0.0376) | -0.0215 | (0.0324) |
| Essential occupation | -0.0706* | (0.0398) | 0.0300 | (0.0375) | 0.0405 | (0.0325) |
| Partner in essential occupation | -0.0920** | (0.0443) | 0.0744* | (0.0430) | 0.0176 | (0.0362) |
| Age | -0.0027 | (0.0024) | 0.00141 | (0.0023) | 0.0013 | (0.0020) |
| Prim. / sec. education | 0.0422 | (0.0665) | 0.0008 | (0.0648) | -0.0429 | (0.0469) |
| Tertiary education | 0.0213 | (0.0449) | -0.0848** | (0.0430) | 0.0636* | (0.0357) |
| Co-resident minor child | 0.0419 | (0.0418) | -0.0413 | (0.0397) | -0.0006 | (0.0336) |
| Workplace autonomy - disagree | 0.1800** | (0.0887) | -0.2950*** | (0.0962) | 0.1150* | (0.0650) |
| Workplace autonomy - agree | 0.2040** | (0.0906) | -0.2860*** | (0.0979) | 0.0820 | (0.0662) |
| Workplace autonomy – not applicable | 0.2210** | (0.104) | -0.2380** | (0.109) | 0.0164 | (0.0746) |
| Partner working fully from home | -0.0223 | (0.0494) | -0.0263 | (0.0463) | 0.0486 | (0.0404) |
| Partner working hybrid | 0.0582 | (0.0611) | -0.0506 | (0.0559) | -0.0075 | (0.0469) |
| Partner working on location,  possibility to work from home | -0.0050 | (0.0749) | -0.0088 | (0.0699) | 0.0139 | (0.0599) |
| Partner not working | 0.2210** | (0.1040) | -0.2380** | (0.1090) | 0.0164 | (0.0746) |

Note: *** p<0.01, ** p<0.05, * p<0.1. Reference categories are women, non-essential occupations, partner in non-essential occupation, vocational education, no minor co-resident children, neutral on statement ‘I can decide where I work’, partner working on location due to the nature of the work.
