# Supplementary material for: Gender inequality in work location, childcare and work-life balance: Phase-specific differences throughout the COVID-19 pandemic
Source: PLoS One. 2024 Jun 25;19(6):e0302633. doi: 10.1371/journal.pone.0302633 (PMC11198899; doi:10.1371/journal.pone.0302633)
Supplement: S28 Table — Note: *** p<0.01, ** p<0.05, * p<0.1. Reference categories are women, non-essential occupations, partner in non-essential occupation, vocational education, no minor co-resident children, neutral on statement ‘I can decide where I work’, partner working on location due to the nature of the work. (DOCX) [file pone.0302633.s029.docx]

**S28 Table. Multinomial logits of work-life balance, including estimated average marginal effects of all covariates in September 2020.**

| September 2020 (n=737) | **Easy** | | **Neutral** | | **Difficult** | |
| --- | --- | --- | --- | --- | --- | --- |
|  | dy/dx | S.E. | dy/dx | S.E. | dy/dx | S.E. |
| Men | 0.0164 | (0.0387) | -0.0183 | (0.0369) | 0.0019 | (0.0253) |
| Essential occupation | -0.0258 | (0.0394) | -0.0108 | (0.0374) | 0.0366 | (0.0262) |
| Partner in essential occupation | -0.0133 | (0.0450) | -0.0686* | (0.0416) | 0.0819** | (0.0339) |
| Age | 0.0034 | (0.0024) | -0.0034 | (0.0023) | 0.0000 | (0.0015) |
| Prim. / sec. education | 0.0753 | (0.0624) | -0.0592 | (0.0603) | -0.0161 | (0.0346) |
| Tertiary education | 0.0443 | (0.0435) | -0.0941** | (0.0422) | 0.0498* | (0.0270) |
| Co-resident minor child | 0.1010** | (0.0402) | -0.0757* | (0.0391) | -0.0257 | (0.0270) |
| Workplace autonomy - disagree | 0.353*** | (0.0773) | -0.3630*** | (0.0894) | 0.0096 | (0.0616) |
| Workplace autonomy - agree | 0.408*** | (0.0795) | -0.410*** | (0.0909) | 0.0019 | (0.0626) |
| Workplace autonomy – not applicable | 0.253*** | (0.0942) | -0.2910*** | (0.104) | 0.0382 | (0.0744) |
| Partner working fully from home | 0.0342 | (0.0516) | -0.0380 | (0.0495) | 0.0038 | (0.0317) |
| Partner working hybrid | 0.0181 | (0.0565) | -0.0220 | (0.0548) | 0.0038 | (0.0344) |
| Partner working on location,  possibility to work from home | 0.0017 | (0.0615) | -0.0530 | (0.0581) | 0.0512 | (0.0426) |
| Partner not working | -0.0281 | (0.0635) | -0.0675 | (0.0580) | 0.0956* | (0.0527) |

Note: *** p<0.01, ** p<0.05, * p<0.1. Reference categories are women, non-essential occupations, partner in non-essential occupation, vocational education, no minor co-resident children, neutral on statement ‘I can decide where I work’, partner working on location due to the nature of the work.
