# Supplementary material for: Gender inequality in work location, childcare and work-life balance: Phase-specific differences throughout the COVID-19 pandemic
Source: PLoS One. 2024 Jun 25;19(6):e0302633. doi: 10.1371/journal.pone.0302633 (PMC11198899; doi:10.1371/journal.pone.0302633)
Supplement: S29 Table — Note: *** p<0.01, ** p<0.05, * p<0.1. Reference categories are women, non-essential occupations, partner in non-essential occupation, vocational education, no minor co-resident children, neutral on statement ‘I can decide where I work’, partner working on location due to the nature of the work. (DOCX) [file pone.0302633.s030.docx]

**S29 Table. Multinomial logits of work-life balance, including estimated average marginal effects of all covariates in November 2020.**

| November 2020 (n=633) | **Easy** | | **Neutral** | | **Difficult** | |
| --- | --- | --- | --- | --- | --- | --- |
|  | dy/dx | S.E. | dy/dx | S.E. | dy/dx | S.E. |
| Men | 0.0852** | (0.0425) | -0.0513 | (0.0410) | -0.0340 | (0.0241) |
| Essential occupation | -0.0434 | (0.0416) | 0.0448 | (0.0402) | -0.0014 | (0.0237) |
| Partner in essential occupation | -0.0266 | (0.0460) | 0.0260 | (0.0445) | 0.0007 | (0.0267) |
| Age | 0.0033 | (0.0026) | -0.0036 | (0.0025) | 0.0004 | (0.0014) |
| Prim. / sec. education | -0.0015 | (0.0689) | 0.0584 | (0.0682) | -0.0569* | (0.0317) |
| Tertiary education | 0.0510 | (0.0471) | -0.0399 | (0.0453) | -0.0111 | (0.0288) |
| Co-resident minor child | 0.0950** | (0.0442) | -0.0246 | (0.0426) | -0.0704** | (0.0291) |
| Workplace autonomy - disagree | 0.1560 | (0.1020) | -0.1610 | (0.1030) | 0.0048 | (0.0584) |
| Workplace autonomy - agree | 0.1310 | (0.1040) | -0.1230 | (0.1060) | -0.0084 | (0.0596) |
| Workplace autonomy – not applicable | 0.0557 | (0.1200) | -0.0997 | (0.121) | 0.0440 | (0.0726) |
| Partner working fully from home | 0.0274 | (0.0510) | -0.0240 | (0.0488) | -0.0034 | (0.0297) |
| Partner working hybrid | -0.0350 | (0.0714) | 0.0759 | (0.0703) | -0.0409 | (0.0347) |
| Partner working on location,  possibility to work from home | -0.0342 | (0.0739) | 0.0577 | (0.0724) | -0.0235 | (0.0381) |
| Partner not working | -0.0454 | (0.0675) | 0.0215 | (0.0658) | 0.0239 | (0.0454) |

Note: *** p<0.01, ** p<0.05, * p<0.1. Reference categories are women, non-essential occupations, partner in non-essential occupation, vocational education, no minor co-resident children, neutral on statement ‘I can decide where I work’, partner working on location due to the nature of the work.
