# Supplementary material for: Gender inequality in work location, childcare and work-life balance: Phase-specific differences throughout the COVID-19 pandemic
Source: PLoS One. 2024 Jun 25;19(6):e0302633. doi: 10.1371/journal.pone.0302633 (PMC11198899; doi:10.1371/journal.pone.0302633)
Supplement: S30 Table — Note: *** p<0.01, ** p<0.05, * p<0.1. Reference categories are women, non-essential occupations, partner in non-essential occupation, vocational education, no minor co-resident children, neutral on statement ‘I can decide where I work’, partner working on location due to the nature of the work. (DOCX) [file pone.0302633.s031.docx]

**S30 Table. Multinomial logits of work-life balance, including estimated average marginal effects of all covariates in November 2021.**

| November 2021 (n=634) | **Easy** | | **Neutral** | | **Difficult** | |
| --- | --- | --- | --- | --- | --- | --- |
|  | dy/dx | S.E. | dy/dx | S.E. | dy/dx | S.E. |
| Men | -0.0263 | (0.0419) | 0.0403 | (0.0388) | -0.0140 | (0.0289) |
| Essential occupation | -0.0528 | (0.0424) | 0.0491 | (0.0390) | 0.0037 | (0.0293) |
| Partner in essential occupation | -0.0380 | (0.0458) | -0.0057 | (0.0418) | 0.0438 | (0.0334) |
| Age | 0.0057** | (0.0026) | -0.0019 | (0.0024) | -0.0038** | (0.0018) |
| Prim. / sec. education | -0.0168 | (0.0700) | 0.0176 | (0.0670) | -0.0008 | (0.0462) |
| Tertiary education | 0.0241 | (0.0463) | -0.0531 | (0.0428) | 0.0291 | (0.0315) |
| Co-resident minor child | 0.0333 | (0.0448) | -0.0187 | (0.0415) | -0.0146 | (0.0316) |
| Workplace autonomy - disagree | 0.1560 | (0.1130) | -0.1110 | (0.1150) | -0.0454 | (0.0936) |
| Workplace autonomy - agree | 0.255** | (0.1140) | -0.1980* | (0.1160) | -0.0571 | (0.0943) |
| Workplace autonomy – not applicable | 0.1910 | (0.1250) | -0.2040* | (0.122) | 0.0134 | (0.105) |
| Partner working fully from home | 0.0607 | (0.0587) | -0.0772 | (0.0515) | 0.0165 | (0.0444) |
| Partner working hybrid | 0.00861 | (0.0547) | -0.00677 | (0.0509) | -0.0018 | (0.0378) |
| Partner working on location,  possibility to work from home | 0.0870 | (0.0723) | -0.0146 | (0.0686) | -0.0724* | (0.0386) |
| Partner not working | -0.0373 | (0.0689) | 0.0326 | (0.0649) | 0.0047 | (0.0523) |

Note: *** p<0.01, ** p<0.05, * p<0.1. Reference categories are women, non-essential occupations, partner in non-essential occupation, vocational education, no minor co-resident children, neutral on statement ‘I can decide where I work’, partner working on location due to the nature of the work.
