# Supplementary material for: Gender inequality in work location, childcare and work-life balance: Phase-specific differences throughout the COVID-19 pandemic
Source: PLoS One. 2024 Jun 25;19(6):e0302633. doi: 10.1371/journal.pone.0302633 (PMC11198899; doi:10.1371/journal.pone.0302633)
Supplement: S31 Table — Note: *** p<0.01, ** p<0.05, * p<0.1. Reference categories are women, non-essential occupations, partner in non-essential occupation, vocational education, no minor co-resident children, neutral on statement ‘I can decide where I work’, partner working on location due to the nature of the work. (DOCX) [file pone.0302633.s032.docx]

**S31 Table. Multinomial logits of work-life balance, including estimated average marginal effects of all covariates in April 2022.**

| November 2022 (n=628) | **Easy** | | **Neutral** | | **Difficult** | |
| --- | --- | --- | --- | --- | --- | --- |
|  | dy/dx | S.E. | dy/dx | S.E. | dy/dx | S.E. |
| Men | 0.0403 | (0.0399) | -0.0123 | (0.0372) | -0.0280 | (0.0265) |
| Age | 0.0025 | (0.0026) | -0.0018 | (0.0024) | -0.0007 | (0.0017) |
| Prim. / sec. education | 0.0689 | (0.0681) | -0.0950 | (0.0623) | 0.0262 | (0.0456) |
| Tertiary education | 0.0329 | (0.0468) | -0.0681 | (0.0443) | 0.0352 | (0.0301) |
| Co-resident minor child | 0.1180*** | (0.0440) | -0.0201 | (0.0410) | -0.0975*** | (0.0333) |
| Workplace autonomy - disagree | 0.1660 | (0.1020) | -0.2080* | (0.1070) | 0.0417 | (0.0716) |
| Workplace autonomy - agree | 0.3140*** | (0.1030) | -0.2870*** | (0.1070) | -0.0265 | (0.0703) |
| Workplace autonomy – not applicable | 0.3400*** | (0.1090) | -0.2940*** | (0.1120) | -0.0462 | (0.0734) |
| Partner working fully from home | 0.0123 | (0.0635) | 0.0145 | (0.0607) | -0.0268 | (0.0410) |
| Partner working hybrid | 0.0908* | (0.0520) | -0.1040** | (0.0458) | 0.0133 | (0.0383) |
| Partner working on location,  possibility to work from home | 0.0490 | (0.0694) | 0.0393 | (0.0670) | -0.088** | (0.0351) |
| Partner not working | 0.0390 | (0.0623) | 0.00718 | (0.0594) | -0.0462 | (0.0393) |

Note: *** p<0.01, ** p<0.05, * p<0.1. Reference categories are women, non-essential occupations, partner in non-essential occupation, vocational education, no minor co-resident children, neutral on statement ‘I can decide where I work’, partner working on location due to the nature of the work.
