# Supplementary material for: Gender inequality in work location, childcare and work-life balance: Phase-specific differences throughout the COVID-19 pandemic
Source: PLoS One. 2024 Jun 25;19(6):e0302633. doi: 10.1371/journal.pone.0302633 (PMC11198899; doi:10.1371/journal.pone.0302633)
Supplement: S32 Table — Note: *** p<0.01, ** p<0.05, * p<0.1. Reference categories are mothers, non-essential occupations, spouse in non-essential occupation, vocational education, partner works on location by nature of work, less childcare. (DOCX) [file pone.0302633.s033.docx]

**S32 Table. Robustness check: Multinomial logits of work-life balance, including estimated average marginal effects of all covariates in April 2020, sub-sample of parents with co-resident minor children.**

| April 2020 (N=602) | Easy | | | Neutral | | | Difficult | |
| --- | --- | --- | --- | --- | --- | --- | --- | --- |
|  | Dy/dx | S.E. | Dy/dx | | S.E. | Dy/dx | | S.E. |
| Men | 0.0261 | (0.0431) | 0.0444 | | (0.0424) | -0.0706* | | (0.0381) |
| Essential occupation | -0.0162 | (0.0411) | 0.0027 | | (0.0405) | 0.0135 | | (0.0366) |
| Spouse in essential occupation | -0.0090 | (0.0452) | 0.0356 | | (0.0441) | -0.0266 | | (0.0390) |
| Age | -0.0054 | (0.0045) | 0.00201 | | (0.0044) | 0.0033 | | (0.0041) |
| Prim. / sec. education | 0.0746 | (0.0732) | 0.0065 | | (0.0719) | -0.0811 | | (0.0897) |
| Tertiary education | -0.0129 | (0.0442) | -0.0876** | | (0.0425) | 0.1010** | | (0.0396) |
| Partner works fully from home | 0.0636 | (0.0488) | -0.0608 | | (0.0483) | -0.0028 | | (0.0427) |
| Partner works hybrid | -0.0216 | (0.0688) | -0.0384 | | (0.0652) | 0.0599 | | (0.0541) |
| Partner works on location; can work from home | 0.1230 | (0.0881) | 0.0434 | | (0.0854) | -0.1670* | | (0.0943) |
| Partner not employed | 0.1200* | (0.0671) | 0.0373 | | (0.0663) | -0.1570** | | (0.0687) |
| More childcare | 0.0794 | (0.0727) | -0.1981*** | | (0.0723) | 0.1190** | | (0.0553) |
| Same childcare | 0.104* | (0.0538) | -0.0294 | | (0.0503) | -0.0746* | | (0.0440) |
| Age youngest child | 0.0230*** | (0.0057) | 0.0029 | | (0.0056) | -0.0259*** | | (0.0053) |

Note: *** p<0.01, ** p<0.05, * p<0.1. Reference categories are mothers, non-essential occupations, spouse in non-essential occupation, vocational education, partner works on location by nature of work, less childcare.
