# Supplementary material for: Gender inequality in work location, childcare and work-life balance: Phase-specific differences throughout the COVID-19 pandemic
Source: PLoS One. 2024 Jun 25;19(6):e0302633. doi: 10.1371/journal.pone.0302633 (PMC11198899; doi:10.1371/journal.pone.0302633)
Supplement: S33 Table — Note: *** p<0.01, ** p<0.05, * p<0.1. Reference categories are mothers, non-essential occupations, spouse in non-essential occupation, vocational education, neutral on workplace autonomy, partner works on location by nature of work, less childcare. (DOCX) [file pone.0302633.s034.docx]

**S33 Table. Robustness check: Multinomial logits of work-life balance, including estimated average marginal effects of all covariates in June 2020, sub-sample of parents with co-resident minor children.**

|  | Easy | | Neutral | | Difficult | |
| --- | --- | --- | --- | --- | --- | --- |
| (June 2020, N=492) | Dy/dx | S.E. | Dy/dx | S.E. | Dy/dx | S.E. |
| Men | 0.1320*** | (0.0494) | -0.0961** | (0.0461) | -0.0360 | (0.0397) |
| Essential occupation | -0.0777 | (0.0480) | 0.0424 | (0.0446) | 0.0353 | (0.0386) |
| Spouse in essential occupation | -0.0945* | (0.0534) | 0.0805 | (0.0512) | 0.0140 | (0.0424) |
| Age | -0.0142*** | (0.0050) | 0.0090** | (0.0046) | 0.0052 | (0.0041) |
| Prim. / sec. education | 0.0334 | (0.0801) | 0.0187 | (0.0810) | -0.0521 | (0.0539) |
| Tertiary education | 0.0625 | (0.0520) | -0.1390*** | (0.0496) | 0.0762* | (0.0409) |
| Workplace autonomy - disagree | 0.2130** | (0.1030) | -0.3150*** | (0.1150) | 0.1020 | (0.0845) |
| Workplace autonomy - agree | 0.2120** | (0.1060) | -0.2780** | (0.1170) | 0.0659 | (0.0859) |
| Workplace autonomy - not applicable | 0.2150* | (0.1200) | -0.2000 | (0.1300) | -0.0158 | (0.0926) |
| Partner works fully from home | -0.0400 | (0.0591) | 0.0050 | (0.0542) | 0.0350 | (0.0491) |
| Partner works hybrid | 0.0573 | (0.0698) | -0.0445 | (0.0618) | -0.0127 | (0.0548) |
| Partner works on location; can work from home | -0.0195 | (0.0899) | 0.0442 | (0.0844) | -0.0247 | (0.0688) |
| Partner not employed | -0.0068 | (0.0748) | 0.0661 | (0.0729) | -0.0593 | (0.0565) |
| More childcare | -0.0077 | (0.0629) | 0.0290 | (0.0554) | -0.0213 | (0.0545) |
| Same childcare | -0.0271 | (0.0527) | 0.0976** | (0.0475) | -0.0705 | (0.0432) |
| Age youngest child | 0.0229*** | (0.0066) | -0.0153** | (0.0062) | -0.0077 | (0.0055) |

Note: *** p<0.01, ** p<0.05, * p<0.1. Reference categories are mothers, non-essential occupations, spouse in non-essential occupation, vocational education, neutral on workplace autonomy, partner works on location by nature of work, less childcare.
