# Supplementary material for: Gender inequality in work location, childcare and work-life balance: Phase-specific differences throughout the COVID-19 pandemic
Source: PLoS One. 2024 Jun 25;19(6):e0302633. doi: 10.1371/journal.pone.0302633 (PMC11198899; doi:10.1371/journal.pone.0302633)
Supplement: S34 Table — Note: *** p<0.01, ** p<0.05, * p<0.1. Reference categories are mothers, non-essential occupations, spouse in non-essential occupation, vocational education, neutral on workplace autonomy, partner works on location by nature of work, less childcare. (DOCX) [file pone.0302633.s035.docx]

**S34 Table. Robustness check: Multinomial logits of work-life balance, including estimated average marginal effects of all covariates in September 2020, sub-sample of parents with co-resident minor children.**

| (September 2020, N=516) | Easy | | Neutral | | Difficult | |
| --- | --- | --- | --- | --- | --- | --- |
|  | Dy/dx | S.E. | Dy/dx | S.E. | Dy/dx | S.E. |
| Men | 0.0356 | (0.0491) | -0.0582 | (0.0470) | 0.0226 | (0.0311) |
| Essential occupation | -0.0085 | (0.0468) | 0.0021 | (0.0445) | 0.0063 | (0.0305) |
| Spouse in essential occupation | 0.0031 | (0.0525) | -0.0700 | (0.0488) | 0.0669* | (0.0370) |
| Age | -0.0049 | (0.0050) | 0.0019 | (0.0047) | 0.0030 | (0.0032) |
| Prim. / sec. education | 0.1200 | (0.0763) | -0.0701 | (0.0741) | -0.0503 | (0.0375) |
| Tertiary education | 0.0453 | (0.0516) | -0.0793 | (0.0498) | 0.0341 | (0.0317) |
| Workplace autonomy - disagree | 0.3650*** | (0.0909) | -0.3370*** | (0.1060) | -0.0276 | (0.0759) |
| Workplace autonomy - agree | 0.4260*** | (0.0940) | -0.3880*** | (0.1080) | -0.0378 | (0.0769) |
| Workplace autonomy - not applicable | 0.2770** | (0.1120) | -0.2640** | (0.1230) | -0.0125 | (0.0906) |
| Partner works fully from home | 0.0491 | (0.0610) | -0.0431 | (0.0583) | -0.0060 | (0.0381) |
| Partner works hybrid | 0.0301 | (0.0649) | -0.0111 | (0.0631) | -0.0190 | (0.0368) |
| Partner works on location; can work from home | 0.0091 | (0.0721) | -0.0596 | (0.0680) | 0.0505 | (0.0521) |
| Partner not employed | 0.0253 | (0.0761) | -0.0500 | (0.0707) | 0.0247 | (0.0540) |
| More childcare | -0.0108 | (0.0660) | 0.0243 | (0.0640) | -0.0135 | (0.0450) |
| Same childcare | 0.0814 | (0.0499) | -0.0399 | (0.0475) | -0.0415 | (0.0334) |
| Age youngest child | 0.0177*** | (0.0066) | -0.0106* | (0.0062) | -0.0071* | (0.0042) |

Note: *** p<0.01, ** p<0.05, * p<0.1. Reference categories are mothers, non-essential occupations, spouse in non-essential occupation, vocational education, neutral on workplace autonomy, partner works on location by nature of work, less childcare.
