# Supplementary material for: Gender inequality in work location, childcare and work-life balance: Phase-specific differences throughout the COVID-19 pandemic
Source: PLoS One. 2024 Jun 25;19(6):e0302633. doi: 10.1371/journal.pone.0302633 (PMC11198899; doi:10.1371/journal.pone.0302633)
Supplement: S35 Table — Note: *** p<0.01, ** p<0.05, * p<0.1. Reference categories are mothers, non-essential occupations, spouse in non-essential occupation, vocational education, neutral on workplace autonomy, partner works on location by nature of work, less childcare. (DOCX) [file pone.0302633.s036.docx]

**S35 Table. Robustness check: Multinomial logits of work-life balance, including estimated average marginal effects of all covariates in November 2020, sub-sample of parents with co-resident minor children.**

|  | Easy | | Neutral | | Difficult | |
| --- | --- | --- | --- | --- | --- | --- |
| (November 2020, N=449) | Dy/dx | S.E. | Dy/dx | S.E. | Dy/dx | S.E. |
| Men | 0.0696 | (0.0519) | -0.0069 | (0.0502) | -0.0628** | (0.0286) |
| Essential occupation | -0.0299 | (0.0497) | 0.0274 | (0.0482) | 0.00251 | (0.0260) |
| Spouse in essential occupation | -0.0322 | (0.0539) | 0.0147 | (0.0518) | 0.0175 | (0.0313) |
| Age | -0.0004 | (0.0052) | -0.0040 | (0.0049) | 0.0044 | (0.0027) |
| Prim. / sec. education | -0.1270 | (0.0818) | 0.1360* | (0.0825) | -0.0089 | (0.0433) |
| Tertiary education | 0.0136 | (0.0549) | -0.00908 | (0.0527) | -0.00453 | (0.0299) |
| Workplace autonomy - disagree | 0.0816 | (0.1270) | -0.0106 | (0.1180) | -0.0710 | (0.0879) |
| Workplace autonomy - agree | 0.0576 | (0.1300) | 0.00228 | (0.1210) | -0.0599 | (0.0890) |
| Workplace autonomy - not applicable | -0.0511 | (0.146) | 0.0689 | (0.1380) | -0.0177 | (0.0995) |
| Partner works fully from home | 0.0094 | (0.0598) | -0.0219 | (0.0575) | 0.0125 | (0.0325) |
| Partner works hybrid | -0.0241 | (0.0799) | 0.0698 | (0.0788) | -0.0457 | (0.0287) |
| Partner works on location; can work from home | -0.1090 | (0.0874) | 0.1130 | (0.0869) | -0.0040 | (0.0407) |
| Partner not employed | -0.0456 | (0.0783) | -0.0071 | (0.0747) | 0.0526 | (0.0547) |
| More childcare | -0.0682 | (0.0684) | 0.0718 | (0.0663) | -0.0037 | (0.0355) |
| Same childcare | -0.0499 | (0.0549) | 0.0521 | (0.0526) | -0.00221 | (0.0290) |
| Age youngest child | 0.0170** | (0.0067) | -0.0106 | (0.0065) | -0.0064* | (0.0036) |

Note: *** p<0.01, ** p<0.05, * p<0.1. Reference categories are mothers, non-essential occupations, spouse in non-essential occupation, vocational education, neutral on workplace autonomy, partner works on location by nature of work, less childcare.
