# Supplementary material for: Gender inequality in work location, childcare and work-life balance: Phase-specific differences throughout the COVID-19 pandemic
Source: PLoS One. 2024 Jun 25;19(6):e0302633. doi: 10.1371/journal.pone.0302633 (PMC11198899; doi:10.1371/journal.pone.0302633)
Supplement: S36 Table — Note: *** p<0.01, ** p<0.05, * p<0.1. Reference categories are mothers, non-essential occupations, spouse in non-essential occupation, vocational education, neutral on workplace autonomy, partner works on location by nature of work, less childcare. (DOCX) [file pone.0302633.s037.docx]

**S36 Table. Robustness check: Multinomial logits of work-life balance, including estimated average marginal effects of all covariates in November 2021, sub-sample of parents with co-resident minor children.**

| (November 2021, N=452) | Easy | | Neutral | | Difficult | |
| --- | --- | --- | --- | --- | --- | --- |
|  | Dy/dx | S.E. | Dy/dx | S.E. | Dy/dx | S.E. |
| Men | 0.0159 | (0.0507) | 0.0008 | (0.0470) | -0.0167 | (0.0351) |
| Essential occupation | -0.0732 | (0.0502) | 0.0717 | (0.0463) | 0.0015 | (0.0345) |
| Spouse in essential occupation | -0.0661 | (0.0539) | 0.0008 | (0.0485) | 0.0653 | (0.0411) |
| Age | -0.0133** | (0.0056) | 0.0114** | (0.00506) | 0.0019 | (0.0038) |
| Prim. / sec. education | -0.0299 | (0.0858) | 0.0362 | (0.0847) | -0.0064 | (0.0538) |
| Tertiary education | 0.0292 | (0.0532) | -0.0672 | (0.0491) | 0.0380 | (0.0354) |
| Workplace autonomy - disagree | 0.2280* | (0.1270) | -0.2200* | (0.1330) | -0.0079 | (0.0950) |
| Workplace autonomy - agree | 0.3100** | (0.1290) | -0.2820** | (0.1350) | -0.0281 | (0.0956) |
| Workplace autonomy - not applicable | 0.2680* | (0.1440) | -0.2930** | (0.1440) | 0.0253 | (0.1120) |
| Partner works fully from home | 0.0847 | (0.0677) | -0.1070* | (0.0583) | 0.0227 | (0.0516) |
| Partner works hybrid | 0.0043 | (0.0627) | 0.0011 | (0.0587) | -0.00540 | (0.0422) |
| Partner works on location; can work from home | 0.0593 | (0.0863) | -0.0034 | (0.0824) | -0.0559 | (0.0460) |
| Partner not employed | -0.0358 | (0.0784) | -0.0013 | (0.0728) | 0.0371 | (0.0615) |
| More childcare | 0.0202 | (0.0651) | -0.0268 | (0.0589) | 0.0066 | (0.0499) |
| Same childcare | 0.0389 | (0.0524) | 0.0139 | (0.0483) | -0.0528 | (0.0367) |
| Age youngest child | 0.0290*** | (0.0070) | -0.0221*** | (0.0065) | -0.0069 | (0.0050) |

Note: *** p<0.01, ** p<0.05, * p<0.1. Reference categories are mothers, non-essential occupations, spouse in non-essential occupation, vocational education, neutral on workplace autonomy, partner works on location by nature of work, less childcare.
