# Supplementary material for: Gender inequality in work location, childcare and work-life balance: Phase-specific differences throughout the COVID-19 pandemic
Source: PLoS One. 2024 Jun 25;19(6):e0302633. doi: 10.1371/journal.pone.0302633 (PMC11198899; doi:10.1371/journal.pone.0302633)
Supplement: S37 Table — Note: *** p<0.01, ** p<0.05, * p<0.1. Reference categories are mothers, vocational education, neutral on workplace autonomy, partner works on location by nature of work. (DOCX) [file pone.0302633.s038.docx]

**S37 Table. Robustness check: Multinomial logits of work-life balance, including estimated average marginal effects of all covariates in April 2022, sub-sample of parents with co-resident minor children.**

| (April 2022, N=425) | Easy | | Neutral | | Difficult | |
| --- | --- | --- | --- | --- | --- | --- |
|  | Dy/dx | S.E. | Dy/dx | S.E. | Dy/dx | S.E. |
| Men | 0.1060** | (0.0486) | -0.0195 | (0.0461) | -0.0863*** | (0.0307) |
| Age | -0.0123** | (0.0052) | 0.00782 | (0.0049) | 0.00448 | (0.0033) |
| Prim. / sec. education | -0.0710 | (0.0832) | -0.0118 | (0.0804) | 0.0828 | (0.0569) |
| Tertiary education | 0.0036 | (0.0529) | -0.0737 | (0.0509) | 0.0701** | (0.0278) |
| Workplace autonomy - disagree | 0.1830 | (0.1310) | -0.1240 | (0.1410) | -0.0590 | (0.1160) |
| Workplace autonomy - agree | 0.3080** | (0.1320) | -0.2180 | (0.1410) | -0.0902 | (0.1150) |
| Workplace autonomy - not applicable | 0.3920*** | (0.1380) | -0.2410* | (0.1460) | -0.152 | (0.1150) |
| Partner works fully from home | -0.0133 | (0.0747) | -0.0117 | (0.0721) | 0.0250 | (0.0489) |
| Partner works hybrid | 0.1030* | (0.0608) | -0.1220** | (0.0554) | 0.0187 | (0.0407) |
| Partner works on location; can work from home | 0.1060 | (0.0758) | -0.0200 | (0.0746) | -0.0861*** | (0.0272) |
| Partner not employed | 0.0170 | (0.0746) | -0.0419 | (0.0700) | 0.0249 | (0.0552) |
| More childcare | 0.1300* | (0.0670) | -0.0988 | (0.0603) | -0.0307 | (0.0476) |
| Same childcare | 0.0796 | (0.0531) | -0.0045 | (0.0505) | -0.0751** | (0.0344) |
| Age youngest child | 0.0279*** | (0.0067) | -0.0168*** | (0.0064) | -0.0111** | (0.0045) |

Note: *** p<0.01, ** p<0.05, * p<0.1. Reference categories are mothers, vocational education, neutral on workplace autonomy, partner works on location by nature of work.
