# Supplementary material for: Gender inequality in work location, childcare and work-life balance: Phase-specific differences throughout the COVID-19 pandemic
Source: PLoS One. 2024 Jun 25;19(6):e0302633. doi: 10.1371/journal.pone.0302633 (PMC11198899; doi:10.1371/journal.pone.0302633)
Supplement: Attachment — Submitted filename: Rebuttal letter.docx [file pone.0302633.s039.docx]

Dear Florian Fischer,

We are pleased to read that you and the two anonymous reviewers feel our manuscript has merit. We further appreciate the opportunity to improve the manuscript based on the comments provided. In this rebuttal letter, we respond to each point raised by yourself and the reviewers. Together with our rebuttal letter, we have included a marked-up copy of our manuscript that highlights changes made to the original version (Revised Manuscript with Track Changes) and an unmarked version of our revised paper without tracked changes (Manuscript).

We feel the changes have substantially improved the manuscript and we look forward to hearing from you and the reviewers in due course.

Kind regards, also on behalf of my co-authors,

Mara Yerkes

**Academic editor comments:**

When submitting your revision, we need you to address these additional requirements.

1. Please ensure that your manuscript meets PLOS ONE's style requirements, including those for file naming. The PLOS ONE style templates can be found at

<https://journals.plos.org/plosone/s/file?id=wjVg/PLOSOne_formatting_sample_main_body.pdf> and

<https://journals.plos.org/plosone/s/file?id=ba62/PLOSOne_formatting_sample_title_authors_affiliations.pdf>

Response: We have carefully edited the manuscript to ensure it follows all style requirements. This includes a check of all headings and removing the short title from the title page. We also now first mention the asterisk followed by the pilcrow symbol. We have also uploaded all Figures to PACE to ensure compliance with PLOS ONE formatting.

2. In your Data Availability statement, you have not specified where the minimal data set underlying the results described in your manuscript can be found. PLOS defines a study's minimal data set as the underlying data used to reach the conclusions drawn in the manuscript and any additional data required to replicate the reported study findings in their entirety. All PLOS journals require that the minimal data set be made fully available. For more information about our data policy, please see <http://journals.plos.org/plosone/s/data-availability>.

Upon re-submitting your revised manuscript, please upload your study’s minimal underlying data set as either Supporting Information files or to a stable, public repository and include the relevant URLs, DOIs, or accession numbers within your revised cover letter. For a list of acceptable repositories, please see <http://journals.plos.org/plosone/s/data-availability#loc-recommended-repositories>. Any potentially identifying patient information must be fully anonymized.

Important: If there are ethical or legal restrictions to sharing your data publicly, please explain these restrictions in detail. Please see our guidelines for more information on what we consider unacceptable restrictions to publicly sharing data: <http://journals.plos.org/plosone/s/data-availability#loc-unacceptable-data-access-restrictions>. Note that it is not acceptable for the authors to be the sole named individuals responsible for ensuring data access.

We will update your Data Availability statement to reflect the information you provide in your cover letter.

Response: There are legal restrictions to sharing our data publicly. The data used for our analyses are owned by the LISS panel and stored in the LISS data archive. Any person interested in replicating our analyses can request access to the LISS data archive and download the data. Subsequently, all steps necessary to reproduce the analyses (i.e., the syntax files) with these data files are outlined in .txt files, all of which will be available on the project website [www.cogisnl.eu/dataandsyntax](http://www.cogisnl.eu/dataandsyntax) following acceptance of the article for publication.

3. We notice that your supplementary figures are uploaded with the file type 'Figure'. Please amend the file type to 'Supporting Information'. Please ensure that each Supporting Information file has a legend listed in the manuscript after the references list.

Response: We have amended the title of all supplementary figures and tables to ‘Supporting Information’. We have also ensured that each file has a legend listed in the manuscript after the references list.

Reviewer #1: This study addresses the diachronic evolution of gender differences in work arrangements, childcare division, and work-life balance.
The descriptive findings extend the existing knowledge by covering approximately two years since the outbreak of the pandemic emergency. The topic is highly relevant for the literature addressing gender inequality and COVID-19-related inequalities, and the paper proposes to make a significant contribution to these readerships. However, major revisions are currently required to meet the publication criteria.

Response: We thank the reviewer for recognizing the significant contribution our article can make and its relevance for the literature.

My major points are:

1) INTRODUCTION AND RESEARCH QUESTION: I personally appreciate the review of existing studies on the topic produced over the last few years, how the existing evidence is critically discussed, and how the question about the evolution of existing disparity over the 2020-2022 period emerges. However, the hypotheses appear poorly motivated and are built upon previous works, primarily from the UK. Although the article does not seem to engage in a detailed theoretical discussion, I believe that the authors should discuss the theoretical mechanisms behind the discussed expectations and the theoretical roles of the mentioned moderators. Furthermore, these motivated expectations require adequate contextualization given the country under scrutiny.

Response: In re-reading the article, we agree with the reviewer (and Reviewer 2, see point 6 below) that our research question and hypotheses required clarification. We indeed do not provide a detailed theoretical discussion here, for reasons of brevity but also given the broad empirical scope and aim of the article. We now refer to other publications in which potential theoretical mechanisms are discussed in more detail, while opting for a short (but in our eyes sufficient) discussion of potential theoretical mechanisms behind our expectations for the current analyses as well as the moderators. These theoretical discussions are now provided together with greater contextualization for the Dutch case in a new section (The Potential for Sensemaking and the Dutch Context, p. 7). The additional information on the Dutch context is also provided to provide greater contextualization in relation to our data collection (see point 3, Reviewer 2). Based on these theoretical discussions, we feel it is prudent to note that we are exploring these relationships throughout the pandemic, rather than testing a specific hypothesis. We now state this in the text (see p. 9).

2) NOT CLEAR SAMPLE STRATEGY: I found it difficult to understand who composed the sample. On page 7, the authors specify that the survey was handed to LISS panel members with at least one working member and a co-resident minor child. However, in the analyses, having or not a child became a control variable (or parenthood as a moderator). Descriptive statistics for the variables of interest were missing. Moreover, if the sample can or cannot have children, the analysis of the childcare division necessarily has a sample different from that of the other analyses. This should be discussed and a description of who composes the different samples should be provided.

Response: We thank the reviewer for this comment. We have now clarified the distinction between the original sample (wave 1) and the extension of this sample in wave 2 to include households without minor co-resident children (see pp. 12-13). Furthermore, we have provided descriptive statistics for all variables of interest (see S2-S5 tables).

3) SELECTION IN SAMPLE: The sampling criteria and two outcome variables require the respondents to be in paid employment. As many of the COVID-19 implications, particularly on gender inequalities, have also occurred by affecting (un-)employment chances, you specifically make a selection of individuals that are still employed in each wave. This selection is not incorrect, but it should also be discussed in relation to the composition of this sample between men and women. Finally, is this selection too strong for the analysis of childcare divisions?

Response: We thank the reviewer for raising this point. Indeed, we recognize that for two outcome variables (work location and work-life balance), respondents indeed need to be in paid employment. We also agree that in many country contexts, gender inequalities have been shaped by (un-)employment chances. In our case country of the Netherlands, however, unemployment (particularly for women) was quite low during the pandemic, likely due to the presence of extensive government measures to protect workers. Additionally, respondents in our sample were asked to indicate whether they were out of work due to the pandemic; only a very small percentage (1-3% from waves 1 through 4) of respondents indicated yes. We reference relevant literature and highlight these points in our Methods section (p. 13) to demonstrate that pandemic-based unemployment does not significantly influence our sampling strategy.

Extending on from this, as the occurrence of COVID-19 related unemployment is so low in our sample, and because parents can be partnered with someone who is unemployed or otherwise not working, we do not feel that our selection is too strong for the analysis of childcare divisions. The table with information on the selection for our dependent variables can be found in the Supplemental Information S3 Table.

4) OUTCOME VARIABLES: Although I like the pre-post operationalization of the outcome variables (i.e. what is the difference with the pre-pandemic period), it would be anyway interesting to look at the absolute values of each wave. This is especially true for childcare, as children may grow over two or more years, and changes in the degree of childcare involvement may be driven by the aging of the child. Moreover, one may also consider the construction of a “within-couple disparity” measure for childcare, where the outcome becomes an imbalance (or changes in the balance) between the two partners.

Response: We thank the reviewer for these suggestions. We agree that particularly for childcare, changes in the degree of childcare involvement could be driven by the aging of the child. In other publications (anonymized for the purposes of review), we have indeed looked at the absolute values of each wave as an outcome variable. However, given the complexity of the current manuscript, and the request (point 6 below) to simplify the manuscript rather than add more analyses, we have decided to refer to our work looking at each wave rather than include it here. Similarly, while a “within-couple disparity” measure would be a useful addition if we were looking solely at the division of childcare, as we are focused on a broader analysis of gender differences throughout the pandemic, we do not include it here.

5) CONTROL VARIABLES: In the analyses, other theoretically driven covariates should be considered. The age of the (youngest) child, for instance, is indicative of the required childcare, the number of children also corrects for differential needs between families, and the biological sex of the child also matters for parent-child interaction. Moreover, the partners’ characteristics and health conditions should be considered. Finally, childcare involvement (and particularly paternal childcare involvement) should also be regarded as a control variable in the study of work-life balance (if all the sampled individuals have at least one child, but indeed this is not clear). For men, it may be easier to combine work and life duties if they do not carry-on childcare activities at all, so men with similar childcare burdens across time should be compared.

Response: We thank the reviewer for providing additional suggestions for control variables. We have now included additional partner characteristics (in addition to whether or not the partner has an essential occupation, we now also include partner’s work location). We include the age of the youngest child for our analyses of the division of childcare and the robustness checks on the sub-sample of parents for the work-life balance outcome (see below; S32-S37 Tables).

Although we agree that the biological sex of the child could be important to include as a control for parent-child interaction, given our focus on gender equality more broadly, we do not include this information here. Additionally, we unfortunately do not have information on health conditions across all data points (such information is only available as part of the core studies of LISS, which are collected on an annual basis). We have therefore chosen to not include them here as a control. We also discuss the exclusion of potential con founders in the Limitations and Future Research section of the Discussion (see p. 31-33). Lastly, in our analyses of work-life balance, the sample includes respondents both with and without children. We have now run robustness checks on the sub-sample of parents in order to allow us to include childcare involvement as a covariate when analyzing the outcome of work-life balance. We have chosen to not include an additional interaction between gender and the level of childcare involvement as that is not the focus of our empirical study. Rather, we maintain our focus on the three moderators of parenthood, essential occupation and education. We report our robustness checks at the end of the Results section (see p. 27).

6) RESULTS: Here is the major point of the review. While going through the paper, I felt that there were simply too many aspects under consideration that hampered a smooth reading and understanding of the contribution. First, the three outcomes are only marginally related to each other, sometimes referring to different reference populations, and in the end, they appear to be chosen more for convenience than for specific theoretical reasons. Second, these outcomes are with multiple categories. Even though the multinomial approach is the correct one, the amount of information to be commented on for each analysis (also considering the two/three moderators) is overwhelming, and I took several readings to grasp them. Alternative operationalizations may help simplify the analyses and communication of the results. Third, the discussion of the results is currently not helpful in simplifying the provided information. In the current version, each result is discussed didactically, without any underlying narrative. Besides reconsidering the overly large focus of the contribution, I would suggest rearranging entirely the discussion of the results, for instance, selecting the most relevant ones and grouping them into subsections (i.e., the overall gap at the outbreak, the temporal evolution, the moderation, etc.).

Response: We thank the reviewer for the helpful suggestion on how to re-organize our results section. To address all three of the points raised (6a: relationship between the three outcomes, 6b: the amount of information being communicated, and 6c: the discussion of the results), we have thoroughly revised the Results section in line with the suggestions of the reviewer. We start by presenting pre-pandemic differences between men and women, followed by the temporal evolution of differences (phase-specific), focused on what changed and when. Lastly, we present our moderated analyses focusing on potential drivers of these gender differences. As the re-organization of our Results section was so extensive, we have done this without track changes (see pp. 21-27). The same is true for the Discussion section (see pp. 27-33). We have also revised the front end of the paper to clarify how these three outcomes relate to each other more broadly, in relation to gender inequalities in work, care and wellbeing (see pp. 4-11; point 6a).

Some minor points:

1) Descriptive figures 1, 2, and 3 were not visible in the version for the review and therefore it was not possible to comment.

Response: We apologize for this oversight. The descriptive figures have now been replaced with tables and we have ensured that all tables and figures are uploaded and available for review.

2) Think about the possibility of including interactions in the main text potentially in a graphical way to simplify the understanding of this large set of results.

Response: We thank the reviewer for this suggestion and have now included three figures to present key interactions in the main text.

3) Avoid using causal language as “effect” when discussing the results.

Response: The reviewer correctly points out the need to avoid causal language here and we have adjusted the text accordingly in both the Results (see pp. 21-27) and Discussion sections (see pp. 27-33).

4) Given the repeated cross-sectional design of the analyses, I would avoid stressing the “longitudinal component” of your contribution. The term diachronic seems to be more appropriate.

Response: We agree with the reviewer that it would be better to avoid stressing the longitudinal component too much. We have now focused on the phase-specific nature of our results, which also aligns with our description of the timing of containment measures discussed in the front end of the paper. We feel this is the most suitable discussion of our contribution, rather than diachronic.

5) On the previous point, why did you not exploit the longitudinal nature of the panel? Other studies have done something similar in other contexts (i.e., Zamberlan et al. 2022, although not acknowledged in the literature review).
Zamberlan, A., Gioachin, F., & Gritti, D. (2022). Gender inequality in domestic chores over ten months of the UK COVID-19 pandemic. Demographic Research, 46, 565-580.

Response: We understand the question from the reviewer. Longitudinal analyses would be interesting if we wanted to consider the relationship between change in particular independent variables and our outcome variables. Additionally, a longitudinal perspective would emphasize the change aspect only, without giving sufficient attention to nuanced changes throughout the pandemic. For this reason, we have chosen to work with repeated cross-sectional analyses to show how gender inequality in work, care and wellbeing has changed within and across the pandemic. We also note that the reference to Zamberlan et al. (2022) was included in the first version of our manuscript (Discussion section, line 611, reference 18), where we discuss how our findings nuance the growing evidence base on divisions of childcare (referenced alongside the work of Lyn Craig and Dorndorfer and colleagues). Alongside this reference, we have also added other relevant references to the limited number of longitudinal studies (and other relevant studies) in the Discussion.

6) The section on limitations does not adequately address limitations, other than the fact that it is a study of only one country (which, in my opinion, is not a limitation if the contribution is properly contextualized). Please elaborate further.

Response: We agree with the reviewer that our Limitations paragraph could be further elaborated and have now done so in the Limitations and Future Research section of the Discussion (see p. 31-33), integrating each of the points raised by both Reviewers.

Reviewer #2: The study is interesting and has a potential to make a contribution to better understand of the development of gender inequalities in paid work, childcare and wellbeing throughout the pandemic, especially in later stages. It identifies that there is a need for greater evidence on this and attempts to fill in the evidence gap. However, the manuscript requires major revisions to meet journal requirements.

Response: We thank the reviewer for seeing the potential contribution of our work.

1. Representativeness/selectiveness of the analytical sample
Main concern relates no effort made to compare representativeness of their “final analytical sample” of the underlying population of workers across the time points analysed. Authors state that a representative, probability-based panel was derived, but it is not clear how the response rates, changes in study design as well as subsequence exclusions affected the representatives of the sample and the subsequent conclusions.
Table 1 shows that at some waves less than a half of the sample made it to their analytical sample, but these proportions vary substantially over time. This is concerning for two main reasons. Firstly, previous studies show that women dropped out from employment at higher rates than men in the early stages of the pandemic. Given the sample is selected based on who remains in paid employment, such inequalities are neglected. Secondly, cross sectional comparison over time can only be meaningful if the same individuals are compared. If this is not possible efforts should be made to ensure that the final analytical sample has the same characteristics over time points compared and is representative of the population of interest. Table S2 compares the samples in terms of the proportion of female but I would be useful to know how the samples compare in terms of the other covariates as well.

Response: We thank the reviewer for these comments concerning our sample and representativeness. Indeed, attrition is an issue in any longitudinal panel dataset. Particularly in the pandemic context, in which women exited employment more often than men in some countries, this issue needs to be discussed. As we note in point 3 for Reviewer 1 (see above), however, evidence from the Netherlands differs in this regard, suggesting that women were not more likely than men to exit paid employment. We have now included a reference on this issue in our manuscript as well as a discussion of the low rate of pandemic-related unemployment in our sample (see p. 13).

Regarding the cross-sectional comparison: We have attempted to make the best use of the data by including respondents in the sample even if they did not participate in every single wave. These are, however, the same individuals across time. We have now clarified this point in our Methods section (see pp. 11-15). We have also included more detail on the descriptive statistics for all covariates and dependent variables (see S2-S5 Tables).

2. Missing data strategy
The authors also state that respondents with missing values were excluded, which would further impact how representative the analytical sample is and therefore how meaningful a comparison over time can be made. Perhaps a more elaborate missing data strategy, such as multiple imputations, should be considered?

Response: We thank the reviewer for this comment, and we have now taken a careful look at the missing variables in our analyses. In doing so, we discovered a mistake in our code that was causing the large number of missings (e.g., people whose partner was not employed were erroneously excluded in some waves). Three of the authors have re-read the code to ensure the reported number of missings is correct. Based on these numbers, we chose to not pursue a multiple imputation strategy but rather to continue with listwise deletion (see Supplemental Information, S1 Table and S3 Table) given the potential for an overestimation of effects (c.f. de Waal, Pannekoek, & Scholtus, 2011). We now discuss this strategy as a potential limitation (see p. 31).

3. Contextualisation
The paper is lacking contextualising both in terms of situating the data in the timeline as well as situating the results in the existing literature. Authors present the timeline in S1, but it is not clear to what extent these restrictions could impact on the results. For example, were the restrictions on work different for those in essential and non-essential occupations? The impact of the restriction on work should feature in the discussion of the findings more explicitly. Similarly, the paper identifies several important international studies, but these do not feed through to the discussion section – are the results similar or different to what previous studies have found? If so, why/why not?

Response: The reviewer correctly points out that contextualization is important, both in terms of understanding Dutch restrictions and the potential impact on our results, as well as for understanding our results in relation to the comparative literature. We have now included a new section to discuss theory and the Dutch context (see point 1, Reviewer 1 above). This section includes greater contextualization of our data collection time points in relation to containment measures as well as additional information on containment restrictions, also in relation to those working in essential versus non-essential occupations.

In addition, we have revised our Discussion section to ensure we reference this contextualization as well as to engage more thoroughly with the international literature cited earlier on in the paper and other relevant sources (see pp. 27-33).

4. Comprehensiveness of the work location outcome variable
It is not clear whether workers were allowed to work from home “by choice”. The variable distinguishes between those who: work from home; work from workplace “by choice”; work from workplace “due to the nature of the work”. At the same time the work autonomy variable allows the choice in terms of work location. This appears inconsistent. Furthermore, previous studies on gender inequality suggest that choices men and women make may reflect internalised social norms. For example, men may be more concerned than women with “breadwinner status” than reputational damage related to neglecting childcare responsibilities, which would affect their behaviours. This should be recognised.
Furthermore, “working partially form home” is shown only in the Figure 1, but not modelled as separate category, which appears inconsistent.

Response: We thank the reviewer for noting the need for clarification of the recoding of our work location variable. We agree that ‘choice’ was a poor word selection, particularly given the answer categories of this variable, which only refers to respondents working at their normal workplace with *the possibility* to work from home. We have now corrected this by changing the wording of our re-coding in the paper (pp. 15-16), helping to distinguish this variable from the work autonomy variable. We have further gone through the manuscript and all tables to ensure we do not refer to this as working at the workplace by choice, but rather, that these respondents worked at the workplace while having the possibility to work from home.

In relation to internalized social norms, we fully agree with the reviewer that choices made by men and women reflect these internalized norms. We now address this point in the Discussion (p.30 lines 643-647).

Lastly, we thank the reviewer for noticing the discrepancy in our modeling. We have now corrected this in the revised version.

5. Floor/ceiling effects
In the analyses of childcare responsibilities, authors define whether respondent are doing more/less compared to before the pandemic. At the same time authors state that division of childcare in the Netherlands was significantly gendered prior to the pandemic, with mothers taking on a much greater role in childcare than fathers. This implies that it may have not been possible for mothers to do even more. Were the ceiling and floor effects of such measurement considered? In the discussion it is also sometimes not clear whether the respondents are doing more/less than they used to or more/less than their partners.

Response: We agree with the reviewer that indeed, for those mothers who were already doing all (or most) of the childcare tasks relative to their partner, even if the amount of care work increased for this group, the relative measure does not capture this - a ceiling effect. We have noted the potential for a ceiling effect in the Discussion of our results (see p. 29).

Additionally, we have worked to clarify the text that we are always referring to the *relative* division of childcare, that is, whether respondents are doing more/less than prior to the pandemic, relative to their partner.

There are also several minor issues:
6. The specific hypothesis and research questions should be made more explicit in the introduction.

Response: We thank the reviewer for noting the need for clarification. We have now revised the front end of the manuscript (also in line with point 1 from Reviewer 1, see above; revisions in pp. 4-11 of the manuscript).

7. There are also issues with reporting self-perceived measures retrospectively, as the subjective perception may change over time or people may simply forget/romanticise/misinterpret. These should be acknowledged.

Response: We have now added a sentence on the use of retrospective measures in the Limitations and Future Research section of the Discussion (see p. 31-33).

8. More rationale/explanation for the definition of essential occupation is needed. Are healthcare workers or teachers not considered as essential?

Response: The definition of essential occupations was developed by the government. We used the government-developed list for each wave of data collection. We have now included further information on this government categorization of essential occupations on pp. 17-18.

9. If possible, it would be useful to control for the age of children in the household, as younger children might need more attention.

Response: We agree with the reviewer and have addressed this issue in relation to point #5 from Reviewer 1 (see above).

Once these issues have been addressed, I would recommend the article for publication.

Response: We thank the reviewer for their confidence in our manuscript and hope the revisions outlined here have sufficiently addressed the concerns of the reviewer.
